# Proteomic assessment of serum biomarkers of longevity in older men

**DOI:** 10.1111/acel.13253

**Published:** 2020-10-20

**Authors:** Eric S. Orwoll, Jack Wiedrick, Carrie M. Nielson, Jon Jacobs, Erin S. Baker, Paul Piehowski, Vladislav Petyuk, Yuqian Gao, Tujin Shi, Richard D. Smith, Douglas C. Bauer, Steven R. Cummings, Jodi Lapidus, for the Osteoporotic Fractures in Men Study (MrOS) Research Group

**Affiliations:** ^1^ Oregon Health & Science University Portland OR USA; ^2^ Biological Science Division Pacific Northwest National Laboratory Richland WA USA; ^3^ Department of Chemistry North Carolina State University Raleigh NC USA; ^4^ Departments of Medicine and Epidemiology & Biostatistics University of California San Francisco CA USA; ^5^ California Pacific Medical Center Research Institute San Francisco CA USA

**Keywords:** aging, biomarker, inflammation, longevity, men, proteomics

## Abstract

The biological bases of longevity are not well understood, and there are limited biomarkers for the prediction of long life. We used a high‐throughput, discovery‐based proteomics approach to identify serum peptides and proteins that were associated with the attainment of longevity in a longitudinal study of community‐dwelling men age ≥65 years. Baseline serum in 1196 men were analyzed using liquid chromatography–ion mobility–mass spectrometry, and lifespan was determined during ~12 years of follow‐up. Men who achieved longevity (≥90% expected survival) were compared to those who died earlier. Rigorous statistical methods that controlled for false positivity were utilized to identify 25 proteins that were associated with longevity. All these proteins were in lower abundance in long‐lived men and included a variety involved in inflammation or complement activation. Lower levels of longevity‐associated proteins were also associated with better health status, but as time to death shortened, levels of these proteins increased. Pathway analyses implicated a number of compounds as important upstream regulators of the proteins and implicated shared networks that underlie the observed associations with longevity. Overall, these results suggest that complex pathways, prominently including inflammation, are linked to the likelihood of attaining longevity. This work may serve to identify novel biomarkers for longevity and to understand the biology underlying lifespan.

## Graphical abstract

In a cohort of older men, discovery‐based serum proteomics at baseline revealed protein biomarkers that were robustly associated with the achievement of longevity during ~12 years of subsequent follow‐up. Longevity‐associated proteins were frequently associated with inflammatory pathways.

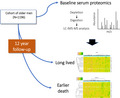

## 1 INTRODUCTION

There have been numerous studies aimed at the identification of prognostic biomarkers of aging outcomes (Barron et al., 2015; Niedernhofer et al., 2017; Sebastiani et al., 2017). Biomarkers could be useful to identify biological processes associated with aging, to identify the likelihood of important health outcomes, and to assess the effectiveness of interventions. Most studies have utilized assays of specific candidate biomarkers that are hypothesized to reflect relevant outcomes (Sanchis‐Gomar et al., 2015), but some have also used more broad ranging analytical approaches aimed at identifying biomarker signatures, for instance using metabolomics (Cheng et al., 2015).

Mass spectrometry (MS)‐based proteomic methods have been successfully adopted for biomarker discovery (Huang et al., 2017), but such proteomic approaches have been limited by technically demanding and time‐consuming methods, and have had inherently low throughput. Previous studies were frequently restricted to relatively small sample sizes that are inadequate to assess associations on a population scale. Newer approaches, such as aptamer‐based or antibody‐based affinity proteomics, allow multiplexing and larger sample sizes but are constrained to the evaluation of candidate proteins (Benson et al., 2019; Gold et al., 2010). We developed high‐throughput and sensitive MS‐based methods that allow a broad, discovery‐based assessment of the serum proteome (Baker et al., 2010, 2014) and have used those methods to interrogate samples from a large longitudinal cohort of older men to identify proteins associated with bone loss and mortality (E. S. Nielson et al., 2017; Orwoll et al., 2018). Similar pipelines for large scale discovery proteomics have been employed in several other pioneering studies (Geyer et al., 2016; Price et al., 2017; Surinova et al., 2015).

We have used discovery proteomics in a 12‐year longitudinal study of older men to identify serum proteins that are associated with longevity and have explored the biological pathways that may be involved in their regulation. Some of these proteins are well documented to be associated with longevity, while others have not been previously reported. These results illustrate the utility of this approach for biomarker discovery, provide candidate protein biomarkers potentially useful to identify individuals who may be long‐lived, and offer insight into the biological basis of longevity.

## 2 RESULTS

### 2.1 Study participants

**Figure 1:**
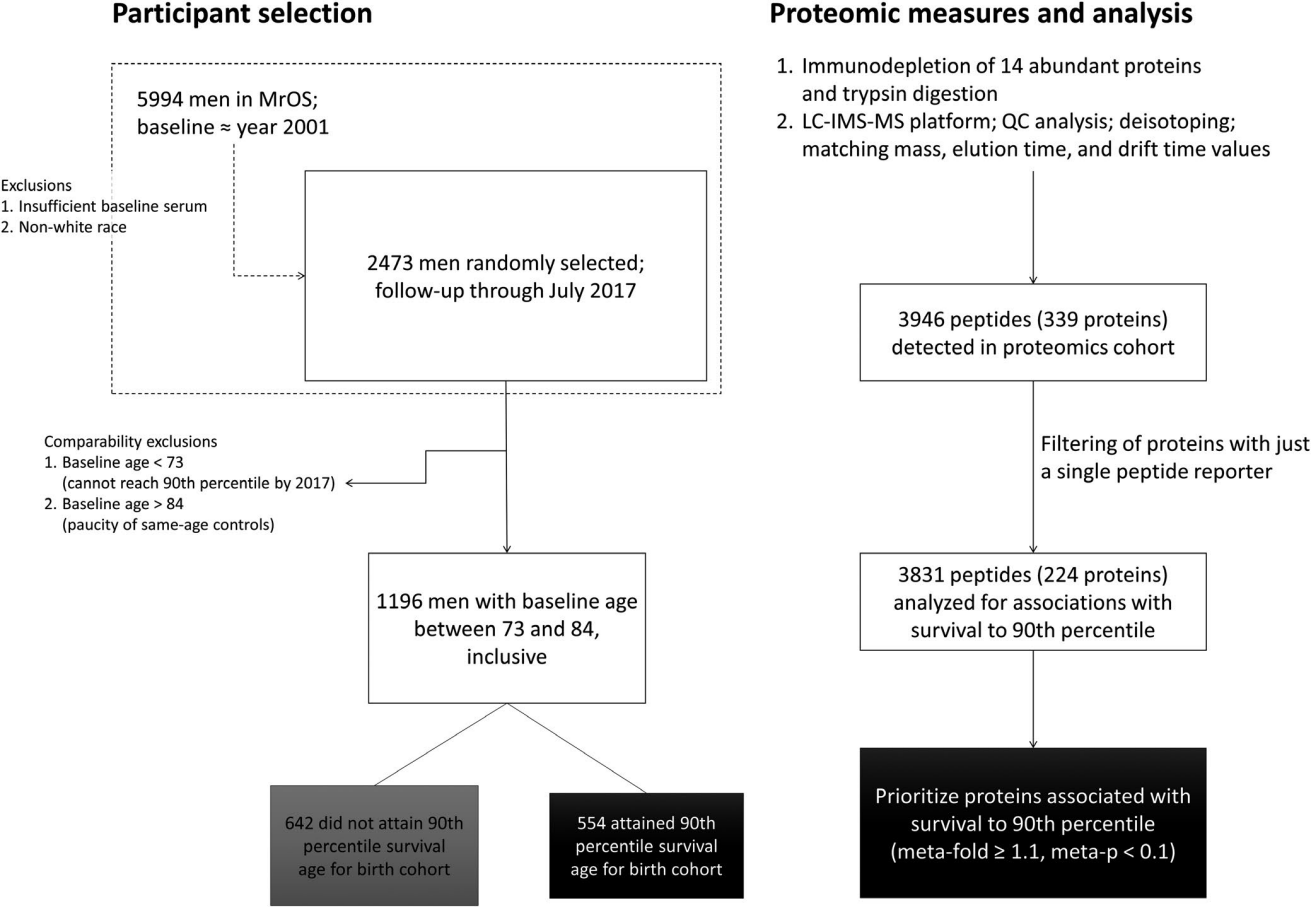
Study overview. Overview of the selection of MrOS participants (left) and the proteomic measurement and analysis workflow (right)

We utilized serum samples and phenotypic data from men ≥65 years enrolled in a large, prospective, longitudinal study (MrOS)(http://mrosdata.sfcc-cpmc.net). Of the entire MrOS cohort (N = 5994), a randomly selected subcohort (N = 2473) had serum proteomic assessments of baseline serum samples and were followed prospectively for 11.9 ± 4.6 years. In these analyses (the analytic cohort), we compared those with proteomic measures who achieved longevity, defined as reaching or exceeding the 90th percentile of expected age for their birth cohort (N = 554), to those who died before achieving longevity (N = 642) (Figure 1). Less than 1% of long‐lived men died within 5 years of baseline, and all men in this group lived at least 3.7 years (the 10th percentile of follow‐up time was 9.1 years). Just 13 (2%) of the shorter‐lived men died within 1 year of baseline, and an additional 30 (5%) in this group died with between 1 year and 2 years of follow‐up. Thus, our study design explicitly mitigated the risk of inadvertently detecting proteins associated with life‐threatening acute illness effects. Potential confounding by age was minimized by requiring complete overlap in baseline age distributions between the two groups (see 4.2 Analytic sample). The characteristics of the overall MrOS cohort, the randomly selected subcohort with proteomic measurements, and the analytic cohort are shown in Table 1. The randomly selected subcohort with proteomic measures was similar to the entire MrOS cohort. In the analytic cohort, the mean age at baseline was 77.4 ± 3.2 years (range 73–84). Generally, these men were similar to the overall MrOS cohort, apart from being slightly older on average due to the age selection criteria. Compared to the shorter‐lived men, the men who achieved longevity were slightly older, had minimally lower BMI, and had slightly better levels of self‐reported health, scores in the physical component of the SF‐12 and scores on the Healthy Aging Index (lower scores are better).

**Table 1:** Cohort characteristics, mean ±SD

	MrOS	Proteomics	Analytic	Long‐lived^a^	Not long‐lived
N	5994	2473	1196	554	642
Age at baseline	73.7 ± 5.9	73.6 ± 5.8	77.4 ± 3.2	78.5 ± 3.1	76.4 ± 2.9
BMI	27.4 ± 3.8	27.4 ± 3.8	27.0 ± 3.5	26.8 ± 3.4	27.2 ± 3.7
Self‐reported health (1–5)^b^	4.2 ± 0.7	4.2 ± 0.7	4.2 ± 0.7	4.2 ± 0.6	4.1 ± 0.7
SF‐12 Physical Component^b^	48.8 ± 10.3	48.9 ± 10.3	47.7 ± 10.8	48.8 ± 9.9	46.7 ± 11.4
SF‐12 Mental Component^b^	55.6 ± 7.0	55.8 ± 6.6	55.6 ± 7.0	55.7 ± 7.0	55.6 ± 7.0
Healthy Aging Index (0–10)^c^	3.0 ± 1.6	2.9 ± 1.6	3.1 ± 1.6	3.0 ± 1.5	3.3 ± 1.7

^a^ To reach or exceed the 90th percentile of expected age for birth cohort.

^b^ Higher score is better.

^c^ Lower score is better.

### 2.2 Proteins associated with longevity

**Figure 2:**
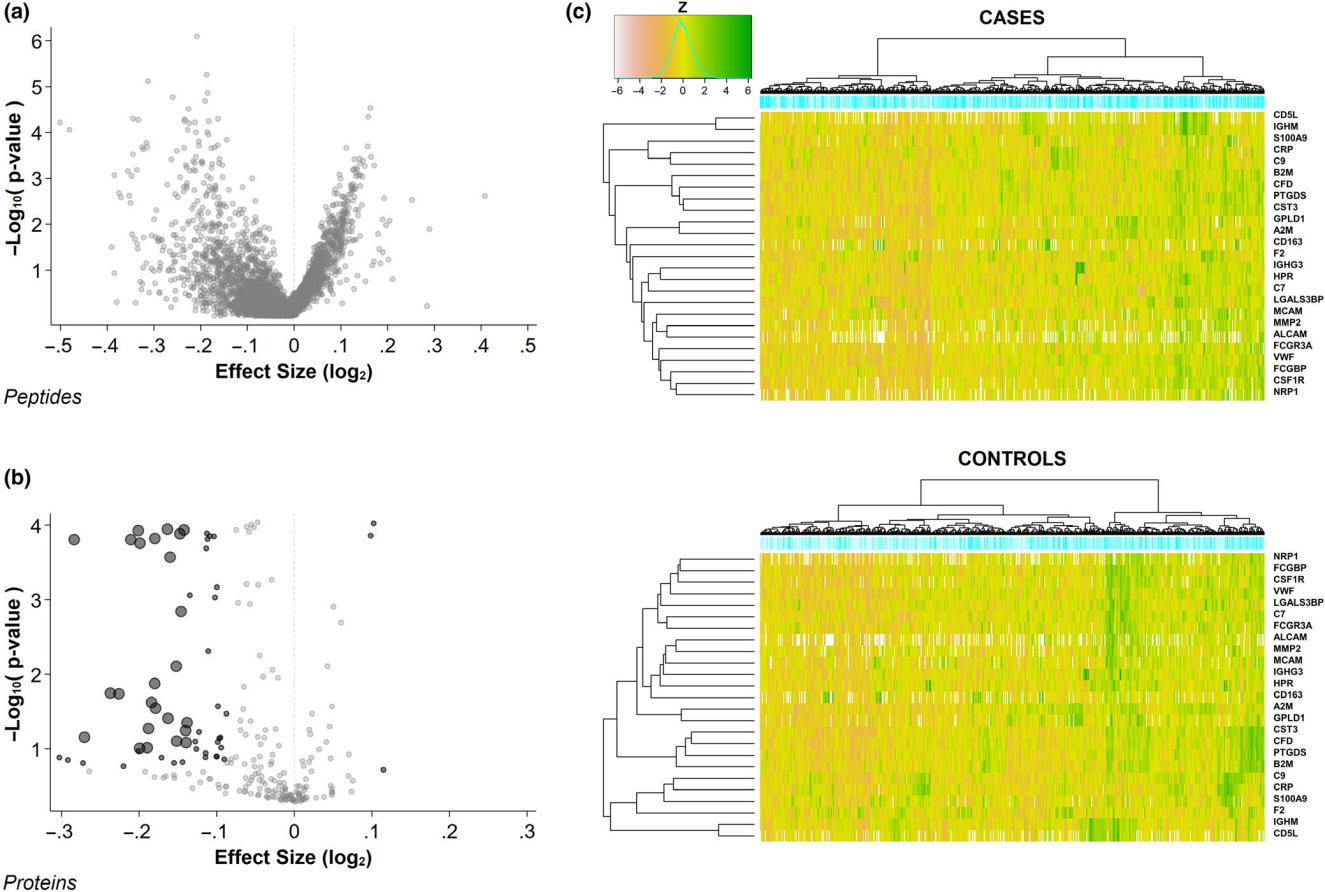
Protein associations with longevity. (a)The associations of 3831 serum peptides identified by MS‐based measurements with the achievement of longevity during observation. Volcano plot of the effect sizes and negative‐log_10_‐transformed p‐values. (b) The associations with longevity of 224 proteins mapping to the 3831 serum peptides. Volcano plot of the effect sizes and log p‐values. Proteins associated with longevity are identified by dark symbols: large black dots =tier 1 proteins (Table 2); small black dots =tier 2 proteins (Supplemental Table 2); small gray dots =nonsignificant proteins. (c) Heatmaps showing the standardized relative abundance of the 25 tier 1 serum proteins associated with longevity in each of the 554 men who achieved longevity during observation (cases, top) and the 642 men who died before achieving longevity (controls, bottom). The z‐scores for all protein associations were precalcuated using the full cohort, so the z‐score values (represented as heatmap colors) are directly comparable between the two panels

We analyzed 3831 serum peptides mapping to 224 proteins. The raw data are available as a MassIVE dataset (accession MSV000085611). Protein identifiers used in the MassIVE files are provided (in the “Symbol” column) in Table S1. The effect sizes of the associations of peptides with longevity are shown in Figure 2a. Protein‐level meta‐analysis of the peptide associations revealed 25 proteins associated with longevity (Table 2), defined as having a meta‐analyzed fold change of at least 1.1 in magnitude and posterior probability of less than 0.1 that the effect is opposite of the estimated direction. An additional 34 proteins (second tier) had significant associations with longevity (Table S2), but with slightly smaller fold changes and slightly higher posterior probabilities of incorrect sign (see 4.4 Statistical analyses). The effect sizes of the protein‐level associations are shown in Figure 2b. All 25 strongly associated proteins (and all but 3 of the 34 second‐tier proteins) were of lower abundance in those men who achieved longevity (fold changes −1.10 to −1.22) than in shorter‐lived men. Key quantitative results from the mass spectrometric data analysis are available in Table S3, including the protein identifiers, number of peptides quantitated, mean relative abundance levels for long‐lived men and controls, fold changes, Bayesian posterior probabilities, and technical coefficients of variation (CVs).

**Table 2:** Proteins with robust absolute fold change >1.1 for longevity

Gene	UniProt	# Peptides	Protein Name	Meta Fold Change	Meta *p*
C9	CO9	19	Complement component C9	−1.217	0.0002
S100A9	S10A9	3	Protein S100‐A9	−1.206	0.0700
CD163	C163A	5	Scavenger receptor cysteine‐rich type 1 protein M130	−1.179	0.0179
CRP	CRP	6	C‐reactive protein	−1.170	0.0183
IGHM	IGHM	19	Immunoglobulin heavy constant mu	−1.157	0.0002
C7	CO7	49	Complement component C7	−1.150	0.0001
FCGR3A	FCG3A	4	Low affinity immunoglobulin gamma Fc region receptor III‐A	−1.148	0.0984
LGALS3BP	LG3BP	14	Galectin‐3‐binding protein	−1.148	0.0002
NRP1	NRP1	3	Neuropilin‐1	−1.140	0.0966
ALCAM	CD166	4	CD166 antigen	−1.139	0.0535
GPLD1	PHLD	7	Phosphatidylinositol‐glycan‐specific phospholipase D	−1.136	0.0239
B2 M	B2MG	7	Beta‐2‐microglobulin	−1.133	0.0133
A2 M	A2MG	21	Alpha‐2‐macroglobulin	−1.133	0.0002
MMP2	MMP2	5	72 kDa type IV collagenase	−1.132	0.0286
VWF	VWF	58	von Willebrand factor	−1.120	0.0001
CSF1R	CSF1R	5	Macrophage colony‐stimulating factor 1 receptor	−1.119	0.0390
HPR	HPTR	13	Haptoglobin‐related protein	−1.117	0.0003
CFD	CFAD	7	Complement factor D	−1.111	0.0078
CD5L	CD5L	6	CD5 antigen‐like	−1.111	0.0788
FCGBP	FCGBP	34	IgGFc‐binding protein	−1.108	0.0001
IGHG3	IGHG3	13	Immunoglobulin heavy constant gamma 3	−1.106	0.0014
F2	THRB	53	Prothrombin	−1.104	0.0001
CST3	CYTC	7	Cystatin‐C	−1.102	0.0569
PTGDS	PTGDS	4	Prostaglandin‐H2 D‐isomerase	−1.101	0.0826
MCAM	MUC18	7	Cell surface glycoprotein MUC18	−1.101	0.0445

The relative abundance levels of the 25 longevity‐associated proteins in the members of the analytic sample are shown in the heatmaps in Figure 2c. Among the men who achieved longevity, there was a large fraction with a pattern of consistently lower abundance levels. That pattern was present in a considerably smaller segment of the men who did not reach longevity, and in the latter group, a larger fraction had a pattern of consistently higher abundance of the longevity proteins.

In a clustering analysis of the complete set of identified serum proteins in the proteomics cohort, there was evidence of 12 clusters of intercorrelated proteins, and those clusters were similar when the clustering was performed separately in both long‐lived men and controls. The 25 longevity‐associated proteins grouped into 5 clusters (Figure S1) showing modest to high levels of pairwise correlation (r = 0.33–0.89) among proteins in each cluster, suggesting that proteins within a cluster may share some underlying regulation.

Although it is difficult to equate tissue levels to circulating protein levels, to explore the tissues that were likely to contribute to the serum proteins associated with longevity, we used studies recently published by Jiang et al. that examined the relative abundance of proteins in human tissues (Jiang L, Wang M, Lin S, Jian R, Li X, Chan JY, Fang H, Dong G, Tang H, Snyder M (2019) A Quantitative Proteome Map of the Human Body. https://www.biorxiv.org/content/10.1101/797373v2). Figure S2 shows the tissues that were most frequently described as having high levels of protein expression of the longevity‐associated proteins. Cardiovascular and neurological tissues were most prominent. The proteins within clusters (above) did not appear to originate more often from unique tissue sources.

### 2.3 Prediction of longevity and mortality

**Figure 3:**
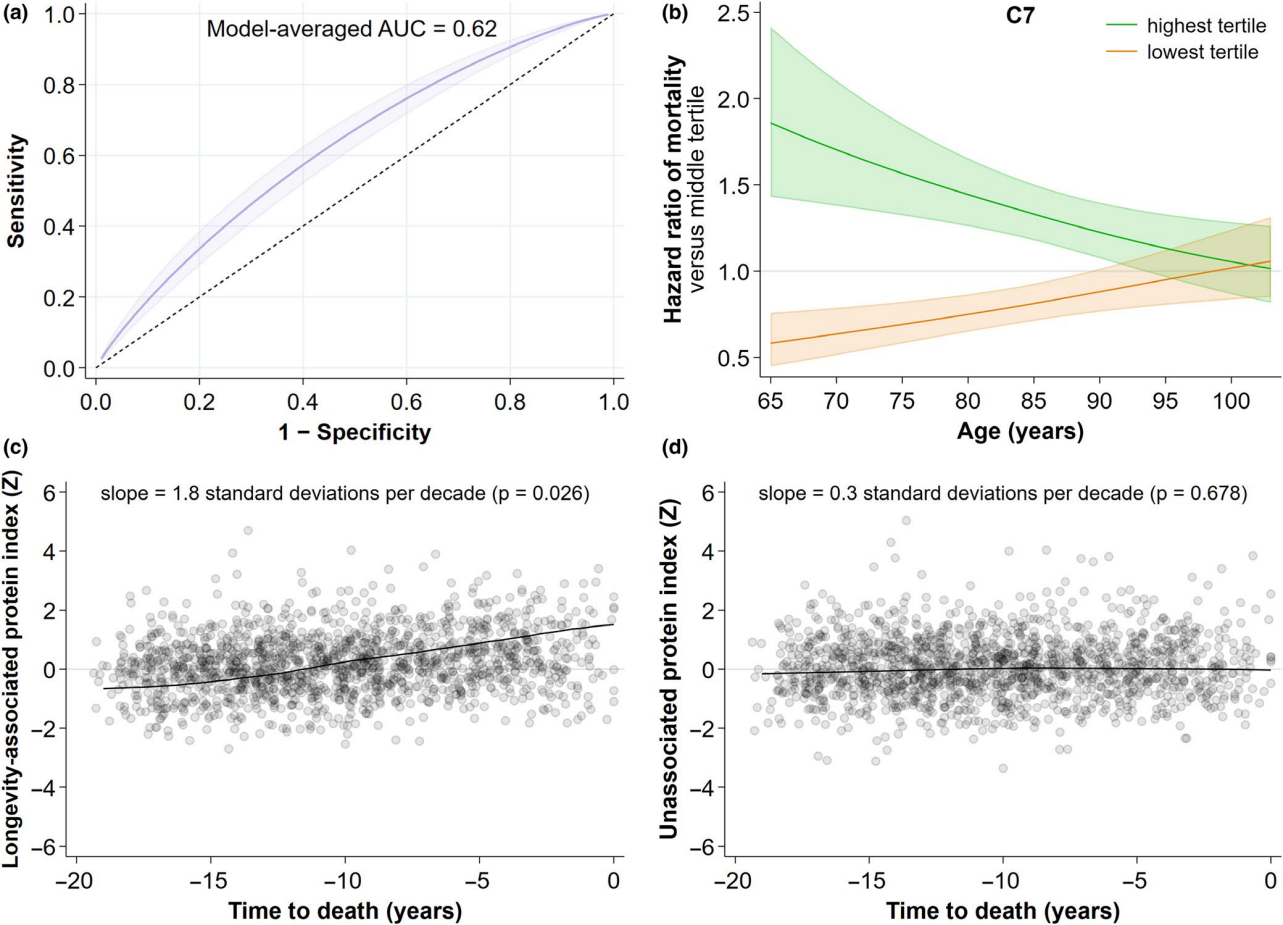
Prediction of longevity and mortality. (a) Receiver operating curve analysis showing the discrimination of long‐lived vs control by a Bayesian model‐averaged classifier comprising a maximally informative subset of 14 of the 25 tier 1 longevity‐associated proteins. (b) The relationship between C7 abundance at baseline age and death rate in the entire proteomic cohort (N = 2473). Shown are the hazard ratios (HR) of death (± 95% CI) for the highest and lowest tertiles of C7 abundance compared to the middle tertile across years of age at baseline. (c) A plot of an abundance index of the 25 tier 1 longevity‐associated proteins (left) as a function of time to death, compared to an abundance index of all 165 measured proteins not associated with longevity (right)

To examine the hypothesis that baseline levels of proteins were predictive of subsequent longevity and mortality, we used several complementary analytical approaches. First, the ability of protein signatures to predict longevity was analyzed using receiver operating characteristics (ROC). All possible combinations of the 25 most robustly associated proteins were evaluated for ability to separate the long‐lived and control groups, and the most informative subset of 14 of the proteins was summarized using Bayesian model averaging where combinations of proteins were weighted by joint posterior model inclusion probability. The Bayesian model‐averaged classifier yielded area under the ROC curve (AUC) of 0.62 (*p* < 0.0001) (Figure 3a). While this finding does not suggest these proteins are clinically useful for the prediction of which men will be long‐lived, it does provide additional evidence of their association with longevity.

Second, when considering either the entire cohort with proteomic measures (N = 2473) or the analytic cohort, higher abundances of each one of these 25 proteins were individually predictive of earlier mortality. Table 3 shows the age‐adjusted hazard ratios of mortality corresponding to standard‐deviation changes in protein abundance for the full proteomics cohort (hazard ratios 1.03–1.32, *p* < 0.0001 for most proteins). An example of the relationships between protein abundance and death rate in the entire proteomics cohort is shown in Figure 3b; men in the highest tertile of C7 levels had a higher risk of death, and those in the lowest tertile a lower risk, compared to those in the middle tertile. As age increased and time to death shortened (see below), the differences were reduced. These analyses yielded similar results in the analytic cohort, including both the long‐lived men and those who died earlier.

**Table 3:** Hazard ratios of longevity‐associated proteins with mortality. The hazard ratios were adjusted for baseline age of the participants

Gene	UniProt	Hazard Ratio	*p*‐value
A2 M	A2MG	1.18	<0.0001
B2 M	B2MG	1.20	<0.0001
CD163	C163A	1.03	0.2891
ALCAM	CD166	1.14	<0.0001
CD5L	CD5L	1.08	0.0109
CFD	CFAD	1.16	<0.0001
C7	CO7	1.32	<0.0001
C9	CO9	1.22	<0.0001
CRP	CRP	1.17	<0.0001
CSF1R	CSF1R	1.15	<0.0001
CST3	CYTC	1.21	<0.0001
FCGR3A	FCG3A	1.14	<0.0001
FCGBP	FCGBP	1.14	<0.0001
HPR	HPTR	1.11	0.0001
IGHG3	IGHG3	1.10	0.0001
LGALS3BP	LG3BP	1.16	<0.0001
MMP2	MMP2	1.13	<0.0001
MCAM	MUC18	1.07	0.0142
IGHM	IGHM	1.08	0.0030
NRP1	NRP1	1.14	<0.0001
GPLD1	PHLD	1.15	<0.0001
PTGDS	PTGDS	1.16	<0.0001
S100A9	S10A9	1.13	<0.0001
F2	THRB	1.11	0.0001
VWF	VWF	1.15	<0.0001

Finally, while the average abundance of the 25 longevity‐associated proteins was lower, at any age, in the long‐lived men than in those who died earlier, in both groups the levels of these proteins tended to be higher in individuals whose time to death was shorter, even after adjustment for baseline age (*p* = 0.026). In contrast, the average abundance of all the other measured proteins that were not associated with longevity was unrelated to the time to eventual death (*p* = 0.68) (Figure 3c). Although protein abundance was assessed at only one time point, these results suggest that an increase in these longevity‐associated proteins heralds impending death.

### 2.4 Proteins associated with longevity are associated with better health status

Centenarians have been reported to have an unexpectedly low burden of adverse health conditions (Gellert et al., 2018). Similarly, in the analytic cohort, lower levels of an overall abundance score summarizing the 25 longevity‐associated proteins (lower score indicates lower protein abundance overall; see Protein abundance summary score in 4.4 Statistical analyses) were significantly associated with a better self‐rated health status (Spearman r = −0.127, *p* < 0.0001), better scores on the SF‐12 physical component index (r = −0.135, *p* < 0.0001), lower (i.e., better) Healthy Aging Index (r = 0.155, *p* < 0.0001), and a lower score on the Fried frailty index (r = 0.192, *p* < 0.0001). The results were the same when the entire proteomics cohort was considered. Moreover, with the exception of the proteins in Cluster 5 (CD5L and IGHM; see Figure S1), each of the protein clusters and all of the proteins in each cluster were individually correlated with these health indices in the same direction as the overall protein score, on average at similar magnitudes but varying (ranging from ~0.02 to ~0.20 in size) depending on the health index and protein. CD5L and IGHM were not correlated with any of the health indices.

### 2.5 Relationship of proteins associated with longevity, mortality and bone loss

**Figure 4:**
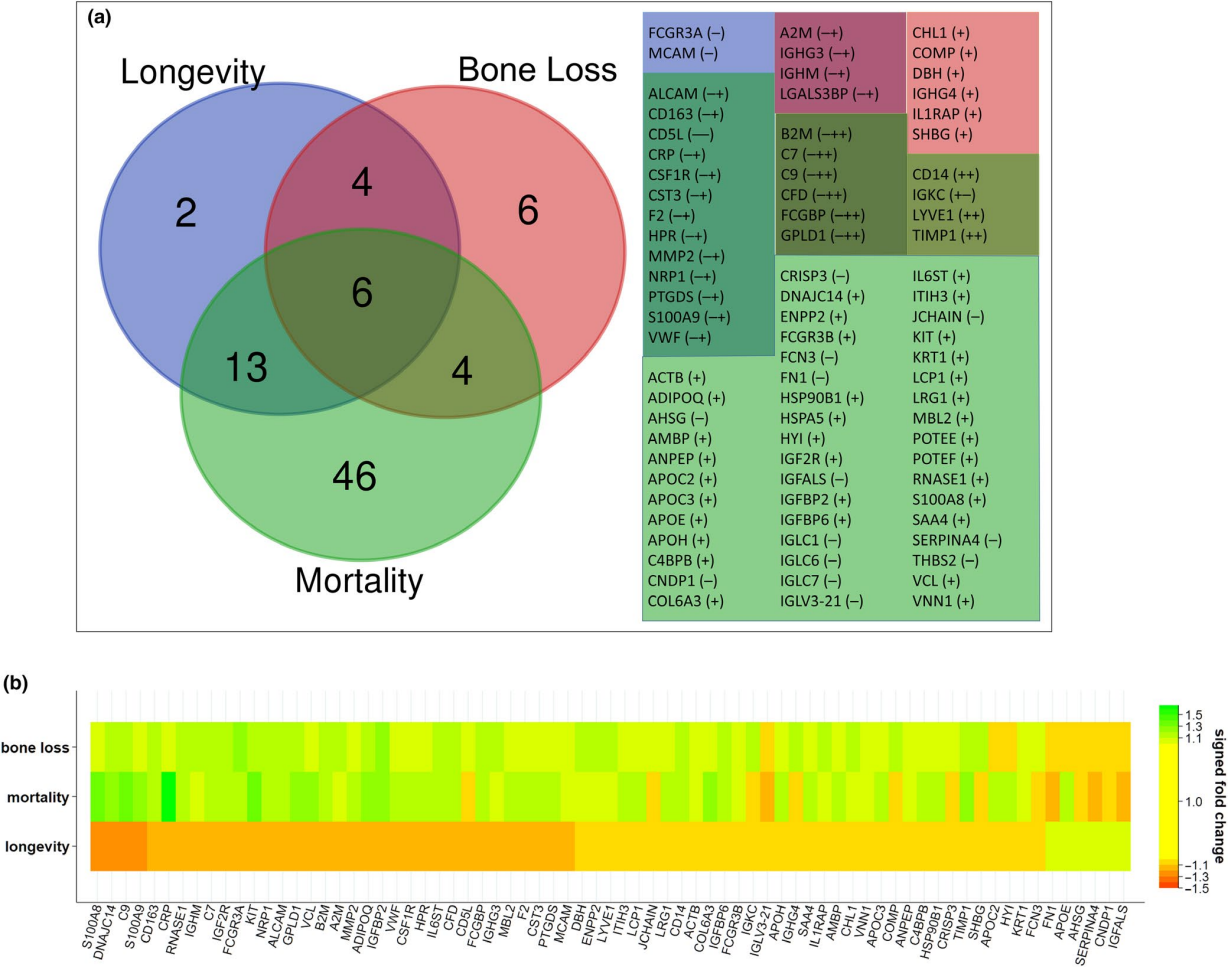
Comparison of protein associations for longevity, mortality, and bone loss. (a) Venn diagram of the overlap of proteins associated with longevity, mortality, and bone loss. The accompanying table lists the overlapping proteins, with protein overlap groups color‐coded to match the regions of the Venn diagram. Shown in parentheses are the directions of protein associations for each phenotype in the order (left to right): longevity, bone loss, mortality. (b) A heatmap of the relative protein abundance of proteins associated with longevity, mortality, and/or bone loss. Shown are the signed fold changes for all proteins that were significantly associated with at least one of the phenotypes using the same criteria for significance that is used in this study (meta‐fold change at least 1.1 in magnitude and meta‐p less than 0.1)

In analyses of the MrOS cohort, we previously reported proteins that are associated with early mortality (E. S. Orwoll et al., 2018) and with accelerated bone loss (Nielson et al., 2017), and we explored to what extent the proteins associated with longevity are also associated with these other two phenotypes. In the Venn diagram in Figure 4a, it is apparent that there is considerable overlap among the proteins associated with longevity, mortality, and accelerated bone loss. However, almost universally, the directions of the associations with longevity are in the opposite direction to those of mortality and bone loss. Figure 4b shows the magnitude and direction of fold changes for each phenotype with each of the proteins referenced in the Venn diagram, and with few exceptions, the protein signature for bone loss and mortality in the top bands of the heatmap is similar and diametrically opposed to that of longevity in the bottom band. To show these relationships in a more quantitative way, we also plotted the values of longevity protein fold changes versus mortality and bone loss fold changes (Figure S3).

### 2.6 Pathway analyses: identification of upstream regulators of longevity‐associated proteins

Ingenuity pathway analysis (IPA) was used to identify upstream regulators and pathways that could be responsible for the proteomic patterns associated with longevity. Upstream regulators are compounds whose biological actions can be directly linked to a protein of interest. The upstream regulators with activation scores |Z|>2 (either activated or inhibited) of the longevity‐associated proteins are shown in Table 4, along with the associated target proteins in our dataset. Of note, accounting for the direction of association in each measured protein that is regulated by the upstream regulator, almost all the upstream regulator pathways highlighted are predicted to be inhibited in long‐lived men. The pathways with high activation scores were all associated with multiple longevity‐associated proteins, and some proteins were members of multiple (>3) upstream regulatory pathways, suggesting a potential convergence of multiple pathways resulting in an altered protein abundance observed in long‐lived men in this study.

**Table 4:** Regulatory pathways for longevity‐associated proteins. Tier 1 proteins associated with longevity appear in boldface, tier 2 proteins appear with neither boldface nor parentheses, and proteins that we did not find significant for longevity but that were linked to the upstream regulators in the IPA knowledge base appear in parentheses. UniProt names and identifiers corresponding to the gene names appearing in the table can be found in Supplemental Table S1

Upstream Regulator	Activation z‐score	Target proteins measured in cohort
Alpha catenin	3.403	C6A3, (IGF1), (IGF2), (IGFBP2), (IGFBP6), (IL6ST), (LUM), **MMP2**, S100A8, **S100A9**, (TIMP1), (VCAM1)
APOE	2.280	ACTB, ADIPOQ, (APOD), (APOE), CD44, CLU, ECM1, (GPX3), (HABP2), (HSP90B1), HSPA5, HSPG2, (IGF1), (IGFBP6), (LRP1), **MMP2**, S100A8, **S100A9**, (SERPINA3), (TIMP1), (VCAM1)
LRP1	2.213	(C1R), (C1S), (LRP1), **MMP2**, (SERPINF1), (SERPING1)
AIRE	−2.000	(AMBP), (IGF2), ITIH3, **S100A9**
PRKCE	−2.000	**CRP**, HSPA5, (IL6ST), (VCAM1)
SOX9	−2.000	(COMP), (KIT), **PTGDS**, (VNN1)
NOS2	−2.058	ADIPOQ, (AZGP1), CD14, CD44, **CFD**, (CP), (ITIH4), **LGALS3BP**, S100A8, (SERPINA3), (TIMP1)
Creb	−2.157	ADIPOQ, CD14, **CD5L**, **CSF1R**, DBH, (IGF1), (KRT1), **MCAM**, **MMP2**, (PCOLCE), (PRG4)
CSF1	−2.159	(APOE), **CD163**, **CSF1R**, **FCGR3A**, (FN1), (HSP90B1), HSPA5, (IGF1), (IL6ST), (PTPRJ), THBS1, (VCAM1)
IL10	−2.166	(APCS), CD14, **CD163**, CD44, **CSF1R**, **FCGR3A**, (IL6ST), **MMP2**, S100A8, (SELL), (TIMP1), (VCAM1)
CHUK	−2.178	CLU, (CP), ENPP2, (IGFBP6), **MMP2**, (NID1), (SOD3), (VCAM1)
GLI1	−2.182	CLU, (FUC2), (IGF1), (IGF2), (IGFBP6), **MMP2**, (PVR), **S100A9**
Vegf	−2.194	**A2 M**, (ANPEP), (APOM), CD44, (CRTAC1), ENPP2, (FN1), (IGFBP3), (IL6ST), LYVE1, **MMP2**, **NRP1**, (PVR), (TIMP1), (VCAM1), **VWF**
F3	−2.195	**F2**, LCP1, **MMP2**, (SERPINC1), (VCAM1)
CXCL12	−2.209	(C5), CD14, CD44, (FN1), (KIT), **MMP2**, THBS1, (TIMP1)
STAT	−2.219	**A2 M**, (AGT), **CRP**, (IL6ST), (SERPINA3), (TIMP1)
HNF4A	−2.313	(A1BG), (AGT), (AHSG), **ALCAM**, (AMBP), (ANPEP), (APCS), (LPA4), (APOC1), (APOC2), (APOC3), (APOE), APOH, (APOM), (C1S), (C2), (C4B), C6, (C8G), (CP), (CPB2), **CRP**, DNAJC14, (F11), (F12), (F13B), (F7), (F9), (FETUB), **GPLD1**, (GSN), (HPX), (HSP90B1), HSPA5, (IGF1), (IL1RAP), (IL6ST), ITIH3, (ITIH4), (KNG1), (LPA), MBL2, (MST1), (PEPD), (PLG), (PON1), PROZ, **PTGDS**, (PTPRG), **S100A9**, (SERPINA10), (SERPINA3), (SERPINA4), (SHBG), (TTR), VTN
CCL2	−2.334	ADIPOQ, (IGF1), **MMP2**, **PTGDS**, (TIMP1), (VCAM1)
IL1B	−2.337	**A2 M**, (APCS), (APOC2), (APOE), **B2 M**, (C1R), CD14, CD44, (CFB), (CP), (CPB2), **CRP**, ENPP2, (FN1), HSPA5, HSPG2, (IGF1), (IGFALS), (IGFBP3), (IGFBP5), (IGFBP6), (IL1RAP), LCP1, **MMP2**, **NRP1**, **PTGDS**, RNASE1, S100A8, **S100A9**, (SELENOP), (SERPINA3), (SERPINF2), (SPARC), THBS1, (TIMP1), (VCAM1)
SREBF1	−2.348	ADIPOQ, (ALDOA), (APOC3), CD14, **CFD**, (CFI), (FN1), (GPX3), HSPA5, **IGHM**, **PTGDS**, (SELENOP), (SERPINA3)
CTNNB1	−2.375	ACTB, ADIPOQ, **ALCAM**, (AOC3), (APOD), C6, CD44, **CFD**, CLU, DBH, ECM1, ENPP2, (FN1), (IGF2), (IGFBP2), (IGFBP5), **IGHM**, (JCHAIN), (KIT), (KRT1), **MMP2**, NCAM1, (PTPRJ), (RBP4), S100A8, (SERPINA3), (TIMP1), (VCAM1)
IL1	−2.395	(APOC3), (APOE), (C2), (CFB), (CP), **CRP**, **CSF1R**, (FN1), (IGF1), (KIT), (LRP1), **MMP2**, (RBP4), **S100A9**, (SAA4), (SELL), (SHBG), (SPARC), (TIMP1), (VCAM1)
CEBPD	−2.408	(AGT), (APOC3), CD14, CLU, (CPB2), **CSF1R**, (IGF1), (IGFBP5)
LDL	−2.429	(APOE), (HSP90B1), HSPA5, (HYOU1), (IGF1), (IGFBP2), (IGFBP3), (LRP1), **MMP2**, S100A8, (VCAM1), (VNN1)
ANGPT2	−2.464	(C1R), (CFB), (FN1), HSPA5, **MMP2**, (PROS1), (SERPING1), (SOD3), THBS1, (VCAM1)
MYD88	−2.550	(APCS), CD14, CD44, HSPA5, (IGF1), (IGFBP5), **MMP2**, S100A8, (TIMP1), (VCAM1)
CEBPA	−2.601	**A2 M**, ADIPOQ, (AGT), (ANPEP), (LPA4), (APOC3), (APOC4), CD14, **CFD**, (CPB2), **CSF1R**, (F9), GGH, **HPR**, HSPA5, (IGF1), (IGFBP3), **NRP1**, (RARRES2), S100A8, **S100A9**, (SERPINF1), THBS1, (VCL)
EGF	−2.629	**ALCAM**, (ANPEP), CD44, CLU, (CPB2), **CSF1R**, (FN1), (IGF1), (IGF2), (IGFALS), (IGFBP2), (IGFBP3), (IGFBP5), **MMP2**, (MST1), NCAM1, **NRP1**, **S100A9**, (SERPINA3), (SPARC), THBS1, (TIMP1), (VCAM1), (VCL)
PRDM1	−2.642	(APOM), CD44, (CFH), **CRP**, ECM1, (F5), (F9), **IGHM**, (JCHAIN), S100A8, **S100A9**, (SELL), (SERPINA3), (TTR)
IL5	−2.763	**A2 M**, (ALDOA), (HSP90B1), HSPA5, **IGHM**, (JCHAIN), (LUM), QSOX1
cytokine	−2.763	CLU, **CRP**, (IGFBP2), **MMP2**, (PON1), (SPARC), (TIMP1), (VCAM1), **VWF**
IL17A	−2.937	ACTB, CD14, **CD163**, **CRP**, **MMP2**, **NRP1**, S100A8, **S100A9**, (SERPIND1), (TIMP1), (VCAM1), **VWF**
IL1A	−2.987	(APOD), CD44, HSPG2, (IGF1), (IGFBP5), (KIT), **MCAM**, **MMP2**, S100A8, **S100A9**, (SERPINA3), (SPARC), (VCAM1)
IL6	−3.053	**A2 M**, (ADAMTS13), (AGT), (ANPEP), (APCS), (APOE), CD14, **CD163**, CD44, **CFD**, (CFH), (CFP), CLU, (COMP), (CP), (CPB2), **CRP**, **CST3**, ENPP2, (F12), (FN1), (GP1BA), (GP5), (HPX), HSPA5, (IGF1), (IGF2), (IGFBP3), (IGFBP5), (IGFBP6), **IGHM**, (IL6ST), (JCHAIN), (KIT), LCAT, (LPA), LRG1, **MMP2**, (PLG), (PON1), (PPBP), **S100A9**, (SAA4), (SERPINA3), (SERPINA7), THBS1, (TIMP1), (TTR), (VCAM1)
ADCYAP1	−3.162	(ATRN), (CRTAC1), ENPP2, (LUM), (MAN1A1), **MCAM**, (SERPINA3), (SERPINF1), (SPARC), (TGFBI)

Several additional analyses supported the relevance of the IPA predictions of upstream regulators. First, serum concentrations of two upstream regulators with high activation scores in our IPA analyses were available from independent ELISA assays (Cauley et al., 2016): IL‐6 and IL‐10, with activation scores −2.834 and −2.166, respectively. As predicted by the IPA, long‐lived men had IL‐6 and IL‐10 concentrations that were lower than other men (22% lower, *p* < 0.001, and 11% lower, *p* = 0.042, respectively). Similarly, serum levels were available for 3 other upstream regulators with less robust activation scores: TNF (activation score = −1.14), TNF receptor 1 (activation score = −1.41), and TNF receptor 2 (activation score = −0.85). Their levels were also, as predicted by IPA, lower in the long‐lived men: 12% lower for TNF (*p* = 0.18), 13% lower for TNF receptor 1 (*p* < 0.001), and 6% lower for TNF receptor 2 (*p* = 0.004). Second, the relative abundance levels of proteins assessed in the present MS‐based analyses that were not associated with longevity, but that were linked by IPA to upstream regulators, were also generally in the directions predicted by IPA. For example, 73% (29 of 40) of the abundance levels of proteins linked to IL‐6 regulation were in the direction predicted, and 83% (10 of 12) of the relationships were as predicted for proteins linked to IL‐10 regulation.

**Figure 5:**
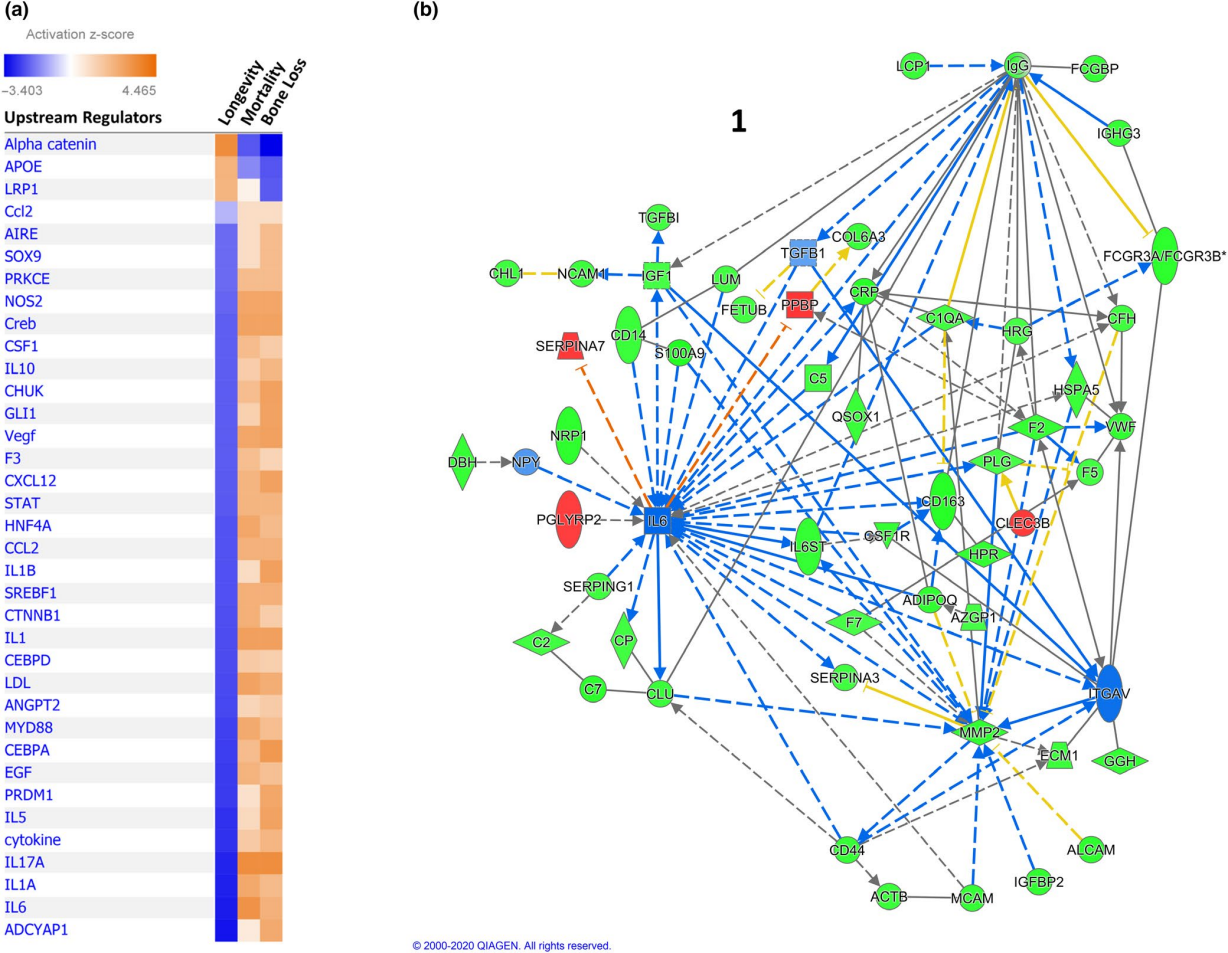
Upstream regulators of proteins associated with longevity. (a) Heatmap showing upstream regulators of longevity‐associated proteins as determined by Ingenuity Pathway Analysis (IPA). Only those regulators with large activation scores (|Z| ≥2) are included. Orange shades indicate IPA‐predicted activation and blue shades indicate predicted inhibition of the regulator. (b) Network analysis (IPA) of the largest cluster (Cluster 1) of intercorrelated serum proteins associated with longevity. To derive these networks, we used IPA network‐building tools in a systematic and algorithmic manner to connect the proteins appearing in the clusters to one another and to annotate their relationships to other closely connected proteins. Green symbols show measured proteins that were decreased in long‐lived men, red symbols measured proteins that were increased, and blue symbols unmeasured proteins or regulators that are predicted by IPA to be inhibited. Blue lines represent inhibitory relationships that were consistent with IPA predictions, orange lines activating relationships consistent with IPA prediction, yellow lines relationships inconsistent with IPA prediction, black lines relationships that exist in the IPA knowledge base but without a prediction, solid lines direct relationships and dashed lines indirect relationships. Arrows indicate directionality of activation, and flat ends show directionality of inhibition. Lines with neither arrows nor flat ends indicate only a general relationship or interaction of the molecules. The names appearing in the figure are IPA gene names, not UniProt identifiers; a mapping of gene names and current UniProt identifiers is in Supplemental Table S1

Using IPA analyses, we also examined how the activation patterns of the upstream regulators of the proteins associated with longevity may differ from those of the mortality and accelerated bone loss phenotypes. In Figure 5a, we show the 35 regulatory pathways with strong activation scores for longevity (activation score |Z|≥2), along with a heatmap displaying the patterns of activation for the regulators across the 3 phenotypes. The directions of activation or inhibition in longevity are uniformly opposite those for bone loss and mortality. Finally, with the hypothesis that the 5 clusters of intercorrelated longevity‐associated proteins described above may reflect shared biological foundations, we examined the upstream regulators that might be involved in regulating the proteins in each cluster. An IPA‐derived network analysis of the largest cluster (including the proteins in the cluster and other biologically associated proteins) is shown in Figure 5b, illustrating the importance of IL6 (which participates in 31 of the 120 connections in the network) and alpha V integrin (IGTAV, participating in 10 connections, including one to IL6) in its regulation. By way of comparison, the average number of connections per gene (beta index) for the network is just 2.14, indicating that the typical amount of connectivity for genes in the network is considerably lower than that displayed by IGTAV and especially IL6. Excluding the connection between them, these two genes account for nearly 25% (39/120) of the total number of edges in the network. Similar network analyses of the other 4 highly intercorrelated clusters containing at least 1 of the 25 longevity proteins are shown in Figure S4. Each of these networks contains at least one hub gene with a node degree 3 to 5 times larger than the beta index for the network. A comprehensive mapping between the gene names used by IPA (and in this paper) and the corresponding UniProt names and identifiers for our 224 measured proteins is provided in Table S1.

## 3 DISCUSSION

High‐throughput proteomic analysis of population‐based study samples provides the opportunity to identify biomarkers for important health outcomes. Using those methods, we identified serum proteins that are associated with longevity in a longitudinal study of older, community‐dwelling men with a long follow‐up period. Further, we used those findings to explore biological pathways that might be involved. The majority of the proteins we identified have been associated with inflammation, although some have multifunctional biological roles and their associations with longevity may reflect other mechanisms. Pathway analyses suggested that several major upstream regulators may be causally responsible for the associations. The proteins and regulatory pathways that are associated with longevity are also associated, but in opposite directions, with the adverse health outcomes of bone loss and mortality. Moreover, late‐life disability and morbidity are lower among people who experience extreme longevity (Hitt et al., 1999). In concert with those findings, the longevity‐associated proteins in this study were associated with several indices of better health status. Finally, we observed a gradual increase in the abundance of longevity‐associated proteins as time to death shortened in both long‐lived and shorter‐lived men. Although the apparent linkage between the abundance of these proteins and death cannot be directly attributed to death *per se*, this finding suggests a shift in underlying biological processes that might be linked to impending death. An understanding of those events would be of obvious interest.

The search for biomarkers of important health outcomes has been a biomedical research priority. Biomarkers can provide tools for prediction and diagnosis, insight into pathophysiology, and targets for the development of therapeutics. To our knowledge, this study represents the largest non‐targeted proteomic effort to discover biomarkers of longevity. Previous proteomic analyses have been limited to smaller numbers of participants, cross‐sectional analyses, and/or to the assessment of specific candidate proteins or other compounds. Some of those studies have used aptamer‐based approaches to scan large numbers of proteins to identify interesting patterns linked to age and health‐related phenotypes and outcomes (Emilsson et al., 2018; Menni et al., 2015; Sun et al., 2018; Tanaka et al., 2018). Our approach offers an unbiased opportunity to identify serum peptides/proteins associated with long life. In fact, our unbiased approach yielded longevity‐associated proteins that were also measured in a recent analysis using a very large aptamer‐based array (Emilsson et al., 2018), but also identified a number (4 of 25; FCGR3A, HPR, FCGBP, MCAM) that were apparently not assessed by that aptamer approach, highlighting the benefit of discovery proteomics.

The fact that many proteins were associated with longevity is not only biologically interesting but also supports the usefulness of population proteomic approaches to identify peptides and proteins of potential usefulness as biomarkers. MS‐based discovery proteomic methods are evolving quickly and more in‐depth measurements should allow a more comprehensive evaluation of similar biomarkers. In addition to the potential value for biomarker development, the identification of factors that are lower in longer‐lived, healthier people may conceivably have eventual therapeutic implications. Parabiosis experiments in animal models suggest that modulation of circulating factors can extend lifespan and improve health (Ashapkin et al., 2020).

The proteins we found to be reduced in long‐lived men strongly reinforce previous findings that implicate a low level of inflammation in the genesis of longevity. For instance, lower CRP was associated with long life in our participants. Similarly, a variety of the longevity‐associated proteins in this study was related to regulation of complement activation, an integral element in both adaptive and innate immune systems that yields the generation of potent inflammatory mediators and cell destruction (Dunkelberger & Song, 2010). Members of the complement cascade that were lower in men who experienced longer life included complement factor D, complement C7, and complement C9. Additional proteins negatively associated with longevity in our analysis are also implicated in inflammatory pathways, including CD166 (activated leukocyte cell adhesion molecule) (Bowen et al., 1995), (Zimmerman et al., 2006), CD5 antigen (Aziz et al., 2015), galectin‐3–binding protein (Yang et al., 2008), macrophage colony‐stimulating factor 1 receptor (Chitu & Stanley, 2006), cell surface glycoprotein (MCAM) (Stevenson et al., 2017), and S100‐A9 (Wang et al., 2018). Adding to these findings, lower levels of several immunoglobulin‐related proteins were associated with longevity (low affinity immunoglobulin gamma Fc region receptor III‐A, immunoglobulin heavy constant mu, immunoglobulin heavy constant gamma 3, IgGFc‐binding protein).

Other longevity‐associated proteins have functions linked to inflammation but are involved in other potentially relevant biological processes as well. Metalloproteinase 9 (72 kDa type IV collagenase) is critical for remodeling of the extracellular matrix (Van den Steen et al., 2002) and cardiovascular physiology (Yabluchanskiy et al., 2013). It is linked to atherosclerosis (Zhu et al., 2017) and heart failure (Meschiari et al., 2018) as well as some elements of the inflammatory response to injury. Scavenger receptor cysteine‐rich type 1 protein (CD163) is exclusively expressed in monocytes and macrophages, is involved in the clearance of hemoglobin/haptoglobin complexes, and may protect tissues from free hemoglobin‐mediated oxidative damage. It is also expressed during the resolution phase of inflammation (Alvarado‐Vazquez et al., 2017; Etzerodt & Moestrup, 2013). Neuropilin is a cell surface receptor that plays important roles in semaphorin and VEGF signaling, and thus in the control of neuronal cell regulation as well as endothelial cell migration and proliferation (Nakamura & Goshima, 2002; Pellet‐Many et al., 2008). It is also expressed in T cells and may help mediated proliferation in response to antigenic stimuli. Beta‐2‐microglobulin (B2 M) is known as a marker of aging and cellular senescence (Althubiti et al., 2014) and is associated with declines in neurogenesis (Villeda et al., 2011). B2 M is the small extracellular immunoglobulin‐like subunit of the major histocompatibility complex (MHC) class I molecule, and its levels are elevated in inflammation, liver or renal dysfunction, some viral infections, and malignancies (Li et al., 2016). Finally, cystatin C levels were lower in men who achieved longevity. Cystatin is a small molecular weight protein and is typically used as a marker of renal function, but higher levels have also been linked to the development of late‐onset Alzheimer's Disease (Chuo et al., 2007) and it has been implicated in diverse aspects of immunity/inflammation and apoptosis (Zi & Xu, 2018).

To provide biological insight into the upstream regulators that could be involved in the generation of the proteomic patterns observed in our data, we utilized IPA analysis that is based on curated predictions from cause–effect relationships reported in the literature (Kramer et al., 2014). IPA analysis is strengthened by the inclusion of knowledge of the direction of pathway interactions (activation or inhibition). In nearly all cases, the activity of the identified regulators is predicted to be inhibited. Although they represent a diversity of biological functions, the list is highly enriched in cytokines and transcription factors which play key roles in the regulation of inflammation and immunity, including IL1α, IL17, CEBPA, PRDM1, IL6, IL1β, IL5, IL10, CXCL12, and others. A variety have been identified as being part of the senescence‐associated secretory phenotype (SASP) (Basisty et al., 2020; Coppe et al., 2008; Matjusaitis et al., 2016), deleterious products released by senescent cells that accumulate with aging (van Deursen, 2014; Kirkland & Tchkonia, 2017). In fact, many of the longevity‐associated proteins (11 of 25) are considered SASP, and four (galectin‐3‐binding protein, CD166 antigen, 72 kDa type IV collagenase, cystatin‐C) are considered "core" SASP—proteins that are consistently stimulated by a variety of senescent stimuli (Basisty et al., 2020). Moreover, there is overlap between the longevity‐associated proteins we have identified (e.g., S100A9, S100A8), or their upstream regulators (e.g., APOE, IL1β, IL6, IL17), and the genes that are differentially expressed in response to caloric restriction and are related to inflammation (Ma et al., 2020). These findings further highlight the strength of the association of reduced inflammation to longevity and may support the hypothesis that lower levels of cell senescence facilitates longer life. Also represented are a number of factors important in cell development or proliferation (including SOX9, ATF4, P53, GLI1, CTNNB1) and metabolism (PRKCE, SREBF1).

Recent work had highlighted the potential importance of circulating protein clusters as biomarkers for important health outcomes (Emilsson et al., 2018), and similarly, we found that the proteins associated with longevity in the current study also clustered. Of the 25 longevity‐associated proteins we identified, 18 were also measured by Emilsson et al. (Emilsson et al., 2018) and 12 were found to be part of clusters in their cohort (AGES). Two of their clusters were enriched in proteins we also found to be associated with longevity. Four of our longevity‐associated proteins (neuropilin, CD166, alpha‐2 microglobulin and 72 kDa type IV collagenase; members of our related clusters 1 and 2, Figure S1) were part of their cluster PM27, a 378‐protein module associated with prevalent heart failure and reduced survival. Three (beta‐2‐microglobulin, complement factor D and cystatin‐C; members of our cluster 3, Figure S1) were part of their cluster PM26, a 390‐protein module that was positively association with prevalent and incident coronary heart disease and heart failure as well as reduced overall survival probability. Clusters such as these may suggest shared biological underpinnings, and our integrative analyses using IPA yielded pathways that appeared to converge on nodes that tied together the longevity‐associated proteins and related proteins and regulators. These analyses may yield targets for additional research evaluation aimed at uncovering causative events related to longevity.

Our analysis has important strengths. It takes advantage of a large, prospective observational study that includes excellent follow‐up and ascertainment of longevity. We analyzed discovery proteomic measures in almost 1200 men, thus representing the largest such experiment available. We utilized very robust statistical methods to link peptides to proteins and to reduce the likelihood of type II error. Several limitations should also be mentioned. The proteomic analysis we performed is limited in terms of sensitivity, but on the other hand, it is relatively comprehensive and we examined a very large number of participants. As our pathway and protein–protein interaction analyses demonstrate, many of the longevity‐associated proteins we report are linked, and although we can implicate major pathways as being associated with longer life, it is more difficult to evaluate the relative importance of each peptide/protein. Since the numbers of minority participants were limited, we could not examine these associations in non‐white men. We did not include women. Observational studies such as ours are limited in their ability to disentangle correlative from the causal factors, and from these analyses, we cannot determine the time of life at which potentially advantageous pathways become associated with longevity. Moreover, we did not include a direct assessment of health, but ultimately it will be important to understand both longevity and disease‐free longevity. Future experimental studies may help to elucidate the relationships among proteins and with outcomes relevant to human health.

In summary, we performed broad based serum proteomic analyses on a large number of older men and describe peptides and proteins that are associated with longevity. Many of the proteins we identified as being reduced in those who were long‐lived are involved in inflammation, and a number were previously found to be linked to early mortality but in the opposite direction. Pathway analyses were highly enriched in regulators of inflammation and immunity, reinforcing the importance of inflammation in the determination of lifespan. These results provide the opportunity to further evaluate these peptides and proteins as biomarkers and highlight the potential importance of the biological pathways they implicate in the origins of long life.

## 4 EXPERIMENTAL PROCEDURES

### 4.1 Study participans

The Study of Osteoporotic Fractures in Men (MrOs) is a prospective observational cohort study of men aged ≥65 years. The design and recruitment have been previously described (Blank et al., 2005; E. Orwoll et al., 2005). Briefly, 5994 community‐dwelling, ambulatory men were recruited from six areas of the US (Birmingham, AL; Minneapolis, MN; Palo Alto, CA; Pittsburgh, PA; Portland, OR; and San Diego, CA) between March 2000 and April 2002. Eligible participants were able to walk without assistance from another person and had not had bilateral hip replacements. Participants or their surrogates were regularly contacted with triannual questionnaires, in part to determine vital status. Rates of follow‐up were high: ~95% of all questionnaires were completed. Reported deaths were confirmed with death certificates. Written informed consent was obtained from all participants. The institutional review board at each study site approved the protocol.

### 4.2 Analytic sample

For the current analysis, 2473 non‐Hispanic white participants were randomly selected from MrOS (Figure 1). Too few non‐white men were enrolled (~10%) to enable analyses of racial or ethnic differences. Within the subcohort of 2473, we selected men who had the potential to achieve longevity, specifically to reach or exceed the 90th percentile of expected age for their birth cohort. That expected age for each birth cohort was defined by an analysis of actuarial life tables from the United States Social Security Administration (see Supplemental Methods). Men who enrolled at ages less than 73 were excluded because they did not have sufficient time to reach the 90th percentile of age for their birth year cohorts. Men who enrolled at ages greater than 84 were excluded because they were already quite close to the 90th percentile of their birth year cohorts; almost none of them failed to reach the 90th percentile during follow‐up, and there were few or no same‐aged controls to compare them to. In order to guard against leverage effects, we required overlap in the age distributions of the long‐lived and not‐long‐lived groups such that each discrete stratum by year of age would contain at least 5 participants from each group. Using these criteria, 1196 men were eligible. Control participants were MrOS subjects with enrollment ages in the selected baseline age range [73‐84] who died during observation (or were lost to follow‐up; 8%) before they reached the 90th percentile of age for their birth year cohort.

### 4.3 Serum proteomic analysis

The proteomics workflow is illustrated in Figure 1 and has been described in detail in (E. S. Orwoll et al., 2018). Briefly, 150 µL of serum from the baseline MrOS visit that had been stored at −80°C since collection was depleted of 14 high‐abundance proteins using IgY14 immunoaffinity depletion columns (Sigma‐Aldrich, St. Louis, MO, USA) and digested with trypsin. A pooled serum from 102 MrOS participants served as technical control of sample processing and analysis; they were embedded throughout the proteomic runs. The tryptic peptide samples were analyzed using a LC‐IMS‐MS platform (Baker et al., 2010, 2014). Specifically, the analytical platform utilized in this work coupled a 1‐m ion mobility drift tube and an Agilent 6224 time‐of‐flight (TOF) mass spectrometer with an upgraded 1.5‐m TOF flight tube providing resolution of ~25,000. The coupled high‐performance LC (HPLC) system was a fully automated in‐house built 4‐column system equipped with in‐house packed capillary columns (30‐cm long having an o.d. of 360 µm, i.d. of 75 µm, and 3‐µm C18 packing material) and operated under a constant flow rate of 1 µL/min (Livesay et al., 2008). Ten µL of each sample was loaded onto a reverse‐phase column and separated over a 58‐min gradient from 100% of mobile phase A (0.5% formic acid in water) to 60% B (0.5% formic acid in 100% acetonitrile). Specifically, the percentage of mobile phase B for 0, 1.2, 12, 51, 58, 59, and 62 minutes was 0, 8, 12, 35, 60, 95, and finally 0% completing the separation/wash cycle. The acquisition range for the MS spectra extended from 100 to 3200 m/z. The details of the platform performance have been described elsewhere (Baker et al., 2010). Detection and quantification of LC‐IMS‐MS features with characteristic (mass, charge, LC elution time, IMS drift time and abundance) was performed using Decon2LS (Jaitly et al., 2009) and FeatureFinder (Crowell et al., 2013) software tools. The detected features were identified by mapping their mass, elution time, and drift time using VIPER software tools (Crowell et al., 2013; Zimmer et al., 2006) and linked to known proteins. Peptide abundances were log_10_ transformed. We removed outliers flagged by a multivariate distance measure. Data normalization was based on estimates of technical variability that were computed from measured abundances of peptides that were detected in all 102 pooled control samples.

### 4.4 Statistical analyses

#### 4.4.1 Peptide and protein‐level associations with long‐lived status

We have published portions of our statistical analysis pipeline previously (Nielson et al., 2017; E. S. Orwoll et al., 2018). We used bias‐corrected estimates from linear regression models to estimate associations between individual peptide abundances and long‐lived status, followed by Bayesian meta‐analyses that combined peptide‐level results to yield associations at the protein level. We then used a resampling procedure to ensure protein‐level estimates were stable.

The linear regression model used normalized log_10_ peptide abundance levels as the dependent variable, and incorporated adjustments only for participant age and the population‐based birth‐cohort cumulative hazard at age to account for differential hazard at the same age in different subcohorts. Measures of body mass index were essentially identical in long‐lived and control groups. The models included indicators for MrOS clinical site (to ensure no variability based on unappreciated differences in study conduct between sites) and an indicator for peptides whose abundance was partially imputed during mass‐spectrometry analysis. The fold difference between peptide abundance in the longevity group and the short‐lived group was estimated as the antilog of $\beta_{1}$ from the following model:

$$\log_{10}peptide\;abundance∼\mu +\beta_{1}alive\;90th+\beta_{2}age+\beta_{3}cumhazard+\left(adjustment\;variables\right)+error$$

Our approach to account for bias in the peptide fold changes caused by missing peptide values was application of the Heckman selection model (Heckman, 1979), the details of which have been previously described (Nielson et al., 2017).

Peptide associations were combined by Bayesian meta‐analysis to yield protein‐level effects ("meta‐effects") based on all peptides mapped to a protein. The sampling variance of each individual peptide estimate was estimated by the squared standard error of the association effect (from the peptide‐level model) and assumed to be a known fixed quantity. We imposed mildly informative Bayesian prior distributions for the mean and variance of the peptide associations, specifically that they were normally distributed with mean 0 and variance 1 on the log_10_ abundance scale, and that the variance was inverse‐gamma distributed with both shape and scale parameters set to 1/100. Estimation was done via Gibbs sampling, and the model was estimated using Markov Chain Monte Carlo (MCMC) with an adaptive burn‐in of 2500 samples and initial MCMC sample size of 10000, times a scaling factor equal to the base‐10 log of the peptide count (or 1, if the peptide count was less than 10), with interim checks for convergence and adaptive expansion of the MCMC sample size if needed. As a sensitivity analysis, the peptide models were re‐estimated with the inclusion of the first 4 GWAS principal components (accounting for >75% of the variance in a GWAS analysis of the MrOS cohort) to adjust for potential genetic biases in the cohort composition and then meta‐analyzed by protein as above, but the impact of this additional adjustment was negligible.

To investigate stability of effect size estimates, we performed a delete‐half jackknife resampling sensitivity analysis based on a bootstrap of 200 jackknife replicates sampled with replacement (Efron, 1994; Shao, 1989). For each replicate, we ran each protein through the entire estimation pipeline and compared the bootstrap distribution of meta‐effect estimates to the full‐cohort estimate. We found that the replicate distribution of effects for proteins represented by at least 2 peptides reliably reproduced the credibility bounds obtained from the full‐cohort meta‐analysis. Singleton peptides were often less stable. Therefore, protein‐level meta‐effects were reported only for the 224 proteins represented by at least 2 measured peptides.

Proteins with differential abundance between the longevity and control groups were selected based on their meta‐effect size and "meta‐p" value (posterior probability that the sign of the meta‐effect is incorrectly estimated). Proteins were prioritized if their bootstrap meta‐p was less than 0.1 and the absolute value of their bootstrap log_10_ meta‐effect was at least 0.041 (corresponding to a fold change of about 1.1 or 0.9). This rule led to the selection of 25 proteins (referred to as "tier 1"; see Table 2). A second tier of 34 proteins with absolute meta‐fold change >1.05 and meta‐*p* < 0.2 that also were in the top third of ranks generated by an empirical Bayes ranking procedure (https://arxiv.org/abs/1312.5776) were tabulated as well (Table S2). It is important to note that these meta‐p values are Bayesian posterior probabilities and do not carry the same interpretation as p‐values. They have already been adjusted for our prior expectations (via the Bayesian prior distributions) and do not require any correction for multiple comparisons.

To facilitate comparisons of longevity proteomic associations with those of the mortality and bone loss phenotypes that we examined in previous papers (Nielson et al., 2017; E. S. Orwoll et al., 2018), we reanalyzed both phenotypes using methods identical to those employed for this study and selected robustly associated proteins using the same selection criteria (absolute meta‐fold change >1.1 and meta‐*p* < 0.1). The selected proteins for these phenotypes are presented in Figure 4a.

#### 4.4.2 Estimates of protein abundance

Protein clustering, receiver operating characteristic (ROC) curves, and correlations with health phenotypes were based on estimates of protein abundance, which were derived via a crossed‐random‐effects model that included all peptides observed for each protein (Nielson et al., 2017). Protein levels for each participant were estimated using predicted values for the total effects (i.e., fixed plus random effects) minus the best linear unbiased prediction of the corresponding random effect for each peptide.

#### 4.4.3 Clustering

To identify clusters of proteins that might share biological regulation, protein abundance estimates were standardized by protein, and pairwise distances between all proteins were calculated using the Gower dissimilarity measure. These distances were clustered hierarchically using Ward's linkage, yielding 12 clusters by the Duda‐Hart stopping rule, 5 of which contained longevity‐associated proteins (from either tier 1 or tier 2).

#### 4.4.4 Protein abundance summary score

Briefly, we standardized the measured abundances within each protein, combined these standardized values across the 25 longevity‐associated proteins (additionally noting the subtotals separately for each cluster), and then standardized the combined total; it is this final overall standardized value that we refer to as the "overall abundance score" above. Scores were additionally calculated for each of the 5 clusters as averages of the (standardized) abundance values of the proteins in each cluster. Hence, a lower score indicates generally lower protein levels. The total protein score was correlated (using Spearman's correlation) with self‐reported health, the SF‐12 physical component, the Healthy Aging Index (Sanders et al., 2014), and the Fried Frailty Index adapted for MrOS (Cawthon et al., 2007; Fried et al., 2001), all measured at the MrOS baseline visit.

#### 4.4.5 Multiprotein longevity signatures

To investigate whether and which combinations of proteins were predictors of long‐lived status, we performed logistic regression modeling and plotted ROC curves. We calculated Mahalanobis distances for participants based on all 25 tier 1 proteins and compared this to alternative calculations of Mahalanobis distances computed from smaller subsets, finding that a set of 14 proteins separated the groups just as well as the full 25. We then fit logistic regression models on all possible combinations of these 14 proteins and computed area under the ROC curve based on predicted values. We used a Bayesian model‐averaging procedure (Burnham KP, Anderson DR. 2002. *Model Selection and Multimodel Inference*:* A Practical Information*‐*Theoretic Approach*, 2e. New York: Springer‐Verlag DOI: 10.1007/b97636) to approximate the average classification performance of multiprotein models and reported this in Figure 3a.

#### 4.4.6 Mortality and death‐proximity associations

We investigated associations between the 25 longevity‐associated proteins and mortality, anticipating that each protein's mortality association would be approximately the inverse of its longevity association. For each protein, we fit a semiparametric time‐to‐event model (using cubic splines), adjusted for participant age, on data from the entire proteomics cohort (N = 2473) as well as the analytic cohort, to obtain a hazard ratio of mortality. Furthermore, to investigate whether proteins were correlated with proximity to death (to calculate the slopes in Figure 3c), we fit a structural equation model (SEM) for the top 25 longevity‐associated proteins, and another for the set of 165 proteins that were not associated with longevity (i.e., including neither tier 1 nor tier 2 proteins; note that tier 2 proteins were not used in either model). The models included a measurement component summarizing the protein abundances into a single factor score. The structural portion of each model assumed the effect of age on protein abundance levels was at least partially mediated by proximity to death. To disentangle the effect of death proximity from the expected aging effect, we used as instrumental variables health status (as measured by the physical component of the SF‐12) and cumulative hazard of death (from US population statistics). Finally, we calculated protein abundance score predictions from the models and performed a kernel‐weighted (Epanechnikov) local polynomial spline smoothing of those predictions along a time‐to‐death axis. The linear association between predicted protein abundance scores and time to death was estimated as the average time derivative of the spline in each instance.

### 4.5 Pathway analyses

We used the Ingenuity Pathway Analysis software (IPA, Spring 2019 release, QIAGEN Inc.; https://digitalinsights.qiagen.com/products-overview/discovery-insights-portfolio/analysis-and-visualization/qiagen-ipa/) to identify networks of interacting proteins associated with longevity, predicted upstream regulators of those associations, and causal networks potentially related to those effects (Kramer et al., 2014). The proteomic dataset was input into ingenuity pathway analysis (IPA) using the Core Analysis platform (Ingenuity Systems, Redwood City, CA) under default settings: Direct and indirect relationships between molecules supported by experimentally observed data were considered, de‐novo networks did not exceed 35 molecules, and all sources of data from human, mouse, and rat studies in the Ingenuity Knowledge Base were considered. IPA provides an upstream regulator analysis to determine likely direct regulators of the proteins in our dataset, designating them as "activated" or "inhibited" based on a z‐score calculated from the fold change directions and magnitudes among the proteins in our data that could be mapped to the regulator. Regulator associations are quantitated by the activation state, including the predicted direction of the associations (activated or inhibited), and the salience of the activation of the putative regulator, as measured by the magnitude of the z‐score. For each cluster (see Clustering), we used IPA network‐building tools in a systematic and algorithmic manner to create networks of genes that according to the IPA knowledge base are closely connected to the proteins comprising each of the 5 protein clusters associated with longevity. Connectivity of genes within each network was assessed by the degree of the gene node (i.e., the number of other genes in the network with a connection to that node) and compared to the overall beta index for the network, which characterizes the average number of connections per node (counting each pair only once).

## CONFLICT OF INTERESTS

All authors have nothing to declare.

## AUTHOR CONTRIBUTIONS

ESO, JW, and JL had full access to data and take responsibility for the integrity of the data and the accuracy of the data analysis. JW, CMN, and JL performed data analysis. ESO, JW, SRC, and JL contributed to study concept and design. ESO, RDS, JJ, and SRC performed data collection/generation. ESO, JW, and JL drafted the manuscript. All authors contributed to data interpretation and manuscript preparation. All authors revised the manuscript content. EO and RDS obtained funding and performed study supervision.

## Data Availability

Data concerning MrOS cohort, including cohort and phenotypic information, are publicly available at http://mrosdata.sfcc-cpmc.net. The raw proteomics data are available as a MassIVE dataset (accession MSV000085611). Protein identifiers used in the MassIVE files are provided (in the “Symbol” column) in Table S1.

## References

[acel13253-bib-0001] Althubiti, M. , Lezina, L. , Carrera, S. , Jukes‐Jones, R. , Giblett, S. M. , Antonov, A. , Barlev, N. , Saldanha, G. S. , Pritchard, C. A. , Cain, K. , & Macip, S. (2014). Characterization of novel markers of senescence and their prognostic potential in cancer. Cell Death & Disease, 5, e1528 10.1038/cddis.2014.489 PMC4260747 25412306

[acel13253-bib-0002] Alvarado‐Vazquez, P. A. , Bernal, L. , Paige, C. A. , Grosick, R. L. , Moracho Vilrriales, C. , Ferreira, D. W. , Ulecia‐Morón, C. , & Romero‐Sandoval, E. A. (2017). Macrophage‐specific nanotechnology‐driven CD163 overexpression in human macrophages results in an M2 phenotype under inflammatory conditions. Immunobiology, 222(8–9), 900‐912. 10.1016/j.imbio.2017.05.011 28545809 PMC5718187

[acel13253-bib-0003] Ashapkin, V. V. , Kutueva, L. I. , & Vanyushin, B. F. (2020). The effects of parabiosis on aging and age‐related diseases. Advances in Experimental Medicine and Biology, 1260, 107‐122. 10.1007/978-3-030-42667-5_5 32304032

[acel13253-bib-0004] Aziz, M. , Holodick, N. E. , Rothstein, T. L. , & Wang, P. (2015). The role of B‐1 cells in inflammation. Immunologic Research, 63(1–3), 153‐166. 10.1007/s12026-015-8708-3 26427372 PMC4651765

[acel13253-bib-0005] Baker, E. S. , Burnum‐Johnson, K. E. , Jacobs, J. M. , Diamond, D. L. , Brown, R. N. , Ibrahim, Y. M. , Orton, D. J. , Piehowski, P. D. , Purdy, D. E. , Moore, R. J. , Danielson, W. F. , Monroe, M. E. , Crowell, K. L. , Slysz, G. W. , Gritsenko, M. A. , Sandoval, J. D. , LaMarche, B. L. , Matzke, M. M. , Webb‐Robertson, B.‐J. , … Smith, R. D. (2014). Advancing the high throughput identification of liver fibrosis protein signatures using multiplexed ion mobility spectrometry. Molecular & Cellular Proteomics: MCP, 13(4), 1119‐1127. 10.1074/mcp.M113.034595 24403597 PMC3977189

[acel13253-bib-0006] Baker, E. S. , Livesay, E. A. , Orton, D. J. , Moore, R. J. , Danielson, W. F. , Prior, D. C. , Ibrahim, Y. M. , LaMarche, B. L. , Mayampurath, A. M. , Schepmoes, A. A. , Hopkins, D. F. , Tang, K. , Smith, R. D. , & Belov, M. E. (2010). An LC‐IMS‐MS platform providing increased dynamic range for high‐throughput proteomic studies. Journal of Proteome Research, 9(2), 997‐1006. 10.1021/pr900888b 20000344 PMC2819092

[acel13253-bib-0007] Barron, E. , Lara, J. , White, M. , & Mathers, J. C. (2015). Blood‐borne biomarkers of mortality risk: systematic review of cohort studies. PLoS One, 10(6), e0127550 10.1371/journal.pone.0127550 26039142 PMC4454670

[acel13253-bib-0008] Basisty, N. , Kale, A. , Jeon, O. H. , Kuehnemann, C. , Payne, T. , Rao, C. , Holtz, A. , Shah, S. , Sharma, V. , Ferrucci, L. , Campisi, J. , & Schilling, B. (2020). A proteomic atlas of senescence‐associated secretomes for aging biomarker development. PLoS Biology, 18(1), e3000599 10.1371/journal.pbio.3000599 31945054 PMC6964821

[acel13253-bib-0009] Benson, M. D. , Ngo, D. , Ganz, P. , & Gerszten, R. E. (2019). Emerging affinity reagents for high throughput proteomics: Trust, but verify. Circulation, 140(20), 1610‐1612. 10.1161/circulationaha.119.039912 31710523 PMC7577374

[acel13253-bib-0010] Blank, J. B. , Cawthon, P. M. , Carrion‐Petersen, M. L. , Harper, L. , Johnson, J. P. , Mitson, E. , & Delay, R. R. (2005). Overview of recruitment for the osteoporotic fractures in men study (MrOS). Contemporary Clinical Trials, 26(5), 557‐568. 10.1016/j.cct.2005.05.005 16085466

[acel13253-bib-0011] Bowen, M. A. , Patel, D. D. , Li, X. , Modrell, B. , Malacko, A. R. , Wang, W. C. , Marquardt, H. , Neubauer, M. , Pesando, J. M. , & Francke, U. (1995). Cloning, mapping, and characterization of activated leukocyte‐cell adhesion molecule (ALCAM), a CD6 ligand. Journal of Experimental Medicine, 181(6), 2213‐2220. 10.1084/jem.181.6.2213 7760007 PMC2192054

[acel13253-bib-0012] Cauley, J. A. , Barbour, K. E. , Harrison, S. L. , Cloonan, Y. K. , Danielson, M. E. , Ensrud, K. E. , Fink, H. A. , Orwoll, E. S. , & Boudreau, R. (2016). Inflammatory Markers and the Risk of Hip and Vertebral Fractures in Men: the Osteoporotic Fractures in Men (MrOS). Journal of Bone and Mineral Research, 31(12), 2129‐2138. 10.1002/jbmr.2905 27371811 PMC5240475

[acel13253-bib-0013] Cawthon, P. M. , Marshall, L. M. , Michael, Y. , Dam, T. T. , Ensrud, K. E. , Barrett‐Connor, E. , & Orwoll, E. S. (2007). Frailty in older men: prevalence, progression, and relationship with mortality. Journal of the American Geriatrics Society, 55(8), 1216‐1223. 10.1111/j.1532-5415.2007.01259.x 17661960

[acel13253-bib-0014] Cheng, S. , Larson, M. G. , McCabe, E. L. , Murabito, J. M. , Rhee, E. P. , Ho, J. E. , Jacques, P. F. , Ghorbani, A. , Magnusson, M. , Souza, A. L. , Deik, A. A. , Pierce, K. A. , Bullock, K. , O’Donnell, C. J. , Melander, O. , Clish, C. B. , Vasan, R. S. , Gerszten, R. E. , & Wang, T. J. (2015). Distinct metabolomic signatures are associated with longevity in humans. Nature Communications, 6, 6791 10.1038/ncomms7791 PMC4396657 25864806

[acel13253-bib-0015] Chitu, V. , & Stanley, E. R. (2006). Colony‐stimulating factor‐1 in immunity and inflammation. Current Opinion in Immunology, 18(1), 39‐48. 10.1016/j.coi.2005.11.006 16337366

[acel13253-bib-0016] Chuo, L. J. , Sheu, W. H. , Pai, M. C. , & Kuo, Y. M. (2007). Genotype and plasma concentration of cystatin C in patients with late‐onset Alzheimer disease. Dementia and Geriatric Cognitive Disorders, 23(4), 251‐257. 10.1159/000100021 17310123

[acel13253-bib-0017] Coppé, J.‐P. , Patil, C. K. , Rodier, F. , Sun, Y. U. , Muñoz, D. P. , Goldstein, J. , Nelson, P. S. , Desprez, P.‐Y. , & Campisi, J. (2008). Senescence‐associated secretory phenotypes reveal cell‐nonautonomous functions of oncogenic RAS and the p53 tumor suppressor. PLoS Biology, 6(12), 2853‐2868. 10.1371/journal.pbio.0060301 19053174 PMC2592359

[acel13253-bib-0018] Crowell, K. L. , Slysz, G. W. , Baker, E. S. , LaMarche, B. L. , Monroe, M. E. , Ibrahim, Y. M. , Payne, S. H. , Anderson, G. A. , & Smith, R. D. (2013). LC‐IMS‐MS Feature Finder: detecting multidimensional liquid chromatography, ion mobility and mass spectrometry features in complex datasets. Bioinformatics, 29(21), 2804‐2805. 10.1093/bioinformatics/btt465 24008421 PMC3799467

[acel13253-bib-0019] Dunkelberger, J. R. , & Song, W. C. (2010). Complement and its role in innate and adaptive immune responses. Cell Research, 20(1), 34‐50. 10.1038/cr.2009.139 20010915

[acel13253-bib-0020] Efron, B. T. R. (1994). An introduction to the bootstrap. Chapman & Hall.

[acel13253-bib-0021] Emilsson, V. , Ilkov, M. , Lamb, J. R. , Finkel, N. , Gudmundsson, E. F. , Pitts, R. , Hoover, H. , Gudmundsdottir, V. , Horman, S. R. , Aspelund, T. , Shu, L. E. , Trifonov, V. , Sigurdsson, S. , Manolescu, A. , Zhu, J. , Olafsson, Ö. , Jakobsdottir, J. , Lesley, S. A. , To, J. , … Gudnason, V. (2018). Co‐regulatory networks of human serum proteins link genetics to disease. Science, 361(6404), 769‐773. 10.1126/science.aaq1327 30072576 PMC6190714

[acel13253-bib-0022] Etzerodt, A. , & Moestrup, S. K. (2013). CD163 and inflammation: biological, diagnostic, and therapeutic aspects. Antioxidants and Redox Signaling, 18(17), 2352‐2363. 10.1089/ars.2012.4834 22900885 PMC3638564

[acel13253-bib-0023] Fried, L. P. , Tangen, C. M. , Walston, J. , Newman, A. B. , Hirsch, C. , Gottdiener, J. , Seeman, T. , Tracy, R. , Kop, W. J. , Burke, G. , & McBurnie, M. A. (2001). Frailty in older adults: evidence for a phenotype. Journals of Gerontology. Series A: Biological Sciences and Medical Sciences, 56(3), M146‐156. 10.1093/gerona/56.3.m146 11253156

[acel13253-bib-0024] Gellert, P. , von Berenberg, P. , Oedekoven, M. , Klemt, M. , Zwillich, C. , Hörter, S. , Kuhlmey, A. , & Dräger, D. (2018). Centenarians differ in their comorbidity trends during the 6 years before death compared to individuals who died in their 80s or 90s. Journals of Gerontology. Series A: Biological Sciences and Medical Sciences, 73(10), 1357‐1362. 10.1093/gerona/glx136 29106492

[acel13253-bib-0025] Geyer, P. E. , Kulak, N. A. , Pichler, G. , Holdt, L. M. , Teupser, D. , & Mann, M. (2016). Plasma proteome profiling to assess human health and disease. Cell Syst, 2(3), 185‐195. 10.1016/j.cels.2016.02.015 27135364

[acel13253-bib-0026] Gold, L. , Ayers, D. , Bertino, J. , Bock, C. , Bock, A. , Brody, E. N. , Carter, J. , Dalby, A. B. , Eaton, B. E. , Fitzwater, T. , Flather, D. , Forbes, A. , Foreman, T. , Fowler, C. , Gawande, B. , Goss, M. , Gunn, M. , Gupta, S. , Halladay, D. , … Zichi, D. (2010). Aptamer‐based multiplexed proteomic technology for biomarker discovery. PLoS One, 5(12), e15004 10.1371/journal.pone.0015004 21165148 PMC3000457

[acel13253-bib-0027] Heckman, J. J. (1979). Sample Selection Bias as a Specification Error. Econometrica, 47(1), 153‐161. 10.2307/1912352

[acel13253-bib-0028] Hitt, R. , Young‐Xu, Y. , Silver, M. , & Perls, T. (1999). Centenarians: the older you get, the healthier you have been. Lancet, 354(9179), 652 10.1016/s0140-6736(99)01987-x 10466675

[acel13253-bib-0029] Huang, Z. , Ma, L. , Huang, C. , Li, Q. , & Nice, E. C. (2017). Proteomic profiling of human plasma for cancer biomarker discovery. Proteomics, 17(6), 10.1002/pmic.201600240 27550791

[acel13253-bib-0030] Jaitly, N. , Mayampurath, A. , Littlefield, K. , Adkins, J. N. , Anderson, G. A. , & Smith, R. D. (2009). Decon2LS: An open‐source software package for automated processing and visualization of high resolution mass spectrometry data. BMC Bioinformatics, 10, 87 10.1186/1471-2105-10-87 19292916 PMC2666663

[acel13253-bib-0031] Kirkland, J. L. , & Tchkonia, T. (2017). Cellular Senescence: A Translational Perspective. Ebiomedicine, 21, 21‐28. 10.1016/j.ebiom.2017.04.013 28416161 PMC5514381

[acel13253-bib-0032] Kramer, A. , Green, J. , Pollard, J. Jr , & Tugendreich, S. (2014). Causal analysis approaches in Ingenuity Pathway Analysis. Bioinformatics, 30(4), 523‐530. 10.1093/bioinformatics/btt703 24336805 PMC3928520

[acel13253-bib-0033] Li, L. , Dong, M. , & Wang, X. G. (2016). The Implication and Significance of Beta 2 Microglobulin: A Conservative Multifunctional Regulator. Chinese Medical Journal (Engl.), 129(4), 448‐455. 10.4103/0366-6999.176084 PMC4800846 26879019

[acel13253-bib-0034] Livesay, E. A. , Tang, K. , Taylor, B. K. , Buschbach, M. A. , Hopkins, D. F. , LaMarche, B. L. , Zhao, R. , Shen, Y. , Orton, D. J. , Moore, R. J. , Kelly, R. T. , Udseth, H. R. , & Smith, R. D. (2008). Fully automated four‐column capillary LC‐MS system for maximizing throughput in proteomic analyses. Analytical Chemistry, 80(1), 294‐302. 10.1021/ac701727r 18044960 PMC2516349

[acel13253-bib-0035] Ma, S. , Sun, S. , Geng, L. , Song, M. , Wang, W. , Ye, Y. , Ji, Q. , Zou, Z. , Wang, S. I. , He, X. , Li, W. , Esteban, C. R. , Long, X. , Guo, G. , Chan, P. , Zhou, Q. I. , Belmonte, J. C. I. , Zhang, W. , Qu, J. , & Liu, G.‐H. (2020). Caloric Restriction Reprograms the Single‐Cell Transcriptional Landscape of Rattus Norvegicus Aging. Cell, 180(5), 984‐1001.e1022. 10.1016/j.cell.2020.02.008 32109414

[acel13253-bib-0036] Matjusaitis, M. , Chin, G. , Sarnoski, E. A. , & Stolzing, A. (2016). Biomarkers to identify and isolate senescent cells. Ageing Research Reviews, 29, 1‐12. 10.1016/j.arr.2016.05.003 27212009

[acel13253-bib-0037] Menni, C. , Kiddle, S. J. , Mangino, M. , Viñuela, A. , Psatha, M. , Steves, C. , Sattlecker, M. , Buil, A. , Newhouse, S. , Nelson, S. , Williams, S. , Voyle, N. , Soininen, H. , Kloszewska, I. , Mecocci, P. , Tsolaki, M. , Vellas, B. , Lovestone, S. , Spector, T. D. , … Valdes, A. M. (2015). Circulating Proteomic Signatures of Chronological Age. Journals of Gerontology. Series A: Biological Sciences and Medical Sciences, 70(7), 809‐816. 10.1093/gerona/glu121 PMC4469006 25123647

[acel13253-bib-0038] Meschiari, C. A. , Jung, M. , Iyer, R. P. , Yabluchanskiy, A. , Toba, H. , Garrett, M. R. , & Lindsey, M. L. (2018). Macrophage overexpression of matrix metalloproteinase‐9 in aged mice improves diastolic physiology and cardiac wound healing after myocardial infarction. American Journal of Physiology. Heart and Circulatory Physiology, 314(2), H224‐h235. 10.1152/ajpheart.00453.2017 29030341 PMC5867652

[acel13253-bib-0039] Nakamura, F. , & Goshima, Y. (2002). Structural and functional relation of neuropilins. Advances in Experimental Medicine and Biology, 515, 55‐69. 10.1007/978-1-4615-0119-0_5 12613543

[acel13253-bib-0040] Niedernhofer, L. J. , Kirkland, J. L. , & Ladiges, W. (2017). Molecular pathology endpoints useful for aging studies. Ageing Research Reviews, 35, 241‐249. 10.1016/j.arr.2016.09.012 27721062 PMC5357461

[acel13253-bib-0041] Nielson, C. M. , Wiedrick, J. , Shen, J. , Jacobs, J. , Baker, E. S. , Baraff, A. , Piehowski, P. , Lee, C. G. , Baratt, A. , Petyuk, V. , McWeeney, S. , Lim, J. Y. , Bauer, D. C. , Lane, N. E. , Cawthon, P. M. , Smith, R. D. , Lapidus, J. , & Orwoll, E. S. (2017). Identification of Hip BMD Loss and Fracture Risk Markers Through Population‐Based Serum Proteomics. Journal of Bone and Mineral Research, 32(7), 1559‐1567. 10.1002/jbmr.3125 28316103 PMC5489383

[acel13253-bib-0042] Orwoll, E. , Blank, J. B. , Barrett‐Connor, E. , Cauley, J. , Cummings, S. , Ensrud, K. , Lewis, C. , Cawthon, P. M. , Marcus, R. , Marshall, L. M. , McGowan, J. , Phipps, K. , Sherman, S. , Stefanick, M. L. , & Stone, K. (2005). Design and baseline characteristics of the osteoporotic fractures in men (MrOS) study–a large observational study of the determinants of fracture in older men. Contemporary Clinical Trials, 26(5), 569‐585. 10.1016/j.cct.2005.05.006 16084776

[acel13253-bib-0043] Orwoll, E. S. , Wiedrick, J. , Jacobs, J. , Baker, E. S. , Piehowski, P. , Petyuk, V. , Gao, Y. , Shi, T. , Smith, R. D. , Bauer, D. C. , Cummings, S. R. , Nielson, C. M. , & Lapidus, J. (2018). High‐throughput serum proteomics for the identification of protein biomarkers of mortality in older men. Aging Cell, 17(2), 10.1111/acel.12717 PMC5847880 29399943

[acel13253-bib-0044] Pellet‐Many, C. , Frankel, P. , Jia, H. , & Zachary, I. (2008). Neuropilins: structure, function and role in disease. Biochemical Journal, 411(2), 211‐226. 10.1042/bj20071639 18363553

[acel13253-bib-0045] Price, N. D. , Magis, A. T. , Earls, J. C. , Glusman, G. , Levy, R. , Lausted, C. , McDonald, D. T. , Kusebauch, U. , Moss, C. L. , Zhou, Y. , Qin, S. , Moritz, R. L. , Brogaard, K. , Omenn, G. S. , Lovejoy, J. C. , & Hood, L. (2017). A wellness study of 108 individuals using personal, dense, dynamic data clouds. Nature Biotechnology, 35(8), 747‐756. 10.1038/nbt.3870 PMC5568837 28714965

[acel13253-bib-0046] Sanchis‐Gomar, F. , Pareja‐Galeano, H. , Santos‐Lozano, A. , Garatachea, N. , Fiuza‐Luces, C. , Venturini, L. , Ricevuti, G. , Lucia, A. , & Emanuele, E. (2015). A preliminary candidate approach identifies the combination of chemerin, fetuin‐A, and fibroblast growth factors 19 and 21 as a potential biomarker panel of successful aging. Age (Dordr), 37(3), 9776 10.1007/s11357-015-9776-y 25911468 PMC4409588

[acel13253-bib-0047] Sanders, J. L. , Minster, R. L. , Barmada, M. M. , Matteini, A. M. , Boudreau, R. M. , Christensen, K. , Mayeux, R. , Borecki, I. B. , Zhang, Q. , Perls, T. , & Newman, A. B. (2014). Heritability of and mortality prediction with a longevity phenotype: the healthy aging index. Journals of Gerontology. Series A: Biological Sciences and Medical Sciences, 69(4), 479‐485. 10.1093/gerona/glt117 PMC3968826 23913930

[acel13253-bib-0048] Sebastiani, P. , Thyagarajan, B. , Sun, F. , Schupf, N. , Newman, A. B. , Montano, M. , & Perls, T. T. (2017). Biomarker signatures of aging. Aging Cell, 16(2), 329‐338. 10.1111/acel.12557 28058805 PMC5334528

[acel13253-bib-0049] Shao, J. (1989). The Efficiency and Consistency of Approximations to the Jackknife Variance Estimators. Journal of the American Statistical Association, 84(405), 114‐119. 10.1080/01621459.1989.10478745

[acel13253-bib-0050] Stevenson, C. , Jiang, D. , Schaefer, N. , Ito, Y. , Berman, R. , Sanchez, A. , & Chu, H. W. (2017). MUC18 regulates IL‐13‐mediated airway inflammatory response. Inflammation Research, 66(8), 691‐700. 10.1007/s00011-017-1050-6 28451734 PMC5612446

[acel13253-bib-0051] Sun, B. B. , Maranville, J. C. , Peters, J. E. , Stacey, D. , Staley, J. R. , Blackshaw, J. , Burgess, S. , Jiang, T. , Paige, E. , Surendran, P. , Oliver‐Williams, C. , Kamat, M. A. , Prins, B. P. , Wilcox, S. K. , Zimmerman, E. S. , Chi, A. N. , Bansal, N. , Spain, S. L. , Wood, A. M. , … Butterworth, A. S. (2018). Genomic atlas of the human plasma proteome. Nature, 558(7708), 73‐79. 10.1038/s41586-018-0175-2 29875488 PMC6697541

[acel13253-bib-0052] Surinova, S. , Choi, M. , Tao, S. , Schuffler, P. J. , Chang, C. Y. , Clough, T. , & Aebersold, R. (2015). Prediction of colorectal cancer diagnosis based on circulating plasma proteins. EMBO Molecular Medicine, 7(9), 1166‐1178. 10.15252/emmm.201404873 26253081 PMC4568950

[acel13253-bib-0053] Tanaka, T. , Biancotto, A. , Moaddel, R. , Moore, A. Z. , Gonzalez‐Freire, M. , Aon, M. A. , Candia, J. , Zhang, P. , Cheung, F. , Fantoni, G. , Semba, R. D. , & Ferrucci, L. (2018). Plasma proteomic signature of age in healthy humans. Aging Cell, 17(5), e12799 10.1111/acel.12799 29992704 PMC6156492

[acel13253-bib-0054] Van den Steen, P. E. , Dubois, B. , Nelissen, I. , Rudd, P. M. , Dwek, R. A. , & Opdenakker, G. (2002). Biochemistry and molecular biology of gelatinase B or matrix metalloproteinase‐9 (MMP‐9). Critical Reviews in Biochemistry and Molecular Biology, 37(6), 375‐536. 10.1080/10409230290771546 12540195

[acel13253-bib-0055] van Deursen, J. M. (2014). The role of senescent cells in ageing. Nature, 509(7501), 439‐446. 10.1038/nature13193 24848057 PMC4214092

[acel13253-bib-0056] Villeda, S. A. , Luo, J. , Mosher, K. I. , Zou, B. , Britschgi, M. , Bieri, G. , Stan, T. M. , Fainberg, N. , Ding, Z. , Eggel, A. , Lucin, K. M. , Czirr, E. , Park, J.‐S. , Couillard‐Després, S. , Aigner, L. , Li, G. E. , Peskind, E. R. , Kaye, J. A. , Quinn, J. F. , … Wyss‐Coray, T. (2011). The ageing systemic milieu negatively regulates neurogenesis and cognitive function. Nature, 477(7362), 90‐94. 10.1038/nature10357 21886162 PMC3170097

[acel13253-bib-0057] Wang, S. , Song, R. , Wang, Z. , Jing, Z. , Wang, S. , & Ma, J. (2018). S100A8/A9 in Inflammation. Frontiers in Immunology, 9, 1298 10.3389/fimmu.2018.01298 29942307 PMC6004386

[acel13253-bib-0058] Yabluchanskiy, A. , Ma, Y. , Iyer, R. P. , Hall, M. E. , & Lindsey, M. L. (2013). Matrix metalloproteinase‐9: Many shades of function in cardiovascular disease. Physiology (Bethesda, Md.), 28(6), 391‐403. 10.1152/physiol.00029.2013 PMC3858212 24186934

[acel13253-bib-0059] Yang, R. Y. , Rabinovich, G. A. , & Liu, F. T. (2008). Galectins: structure, function and therapeutic potential. Expert Reviews in Molecular Medicine, 10, e17 10.1017/s1462399408000719 18549522

[acel13253-bib-0060] Zhu, J.‐J. , Zhao, Q. , Qu, H.‐J. , Li, X.‐M. , Chen, Q.‐J. , Liu, F. , Chen, B.‐D. , & Yang, Y.‐N. (2017). Usefulness of plasma matrix metalloproteinase‐9 levels in prediction of in‐hospital mortality in patients who received emergent percutaneous coronary artery intervention following myocardial infarction. Oncotarget, 8(62), 105809‐105818. 10.18632/oncotarget.22401 29285294 PMC5739681

[acel13253-bib-0061] Zi, M. , & Xu, Y. (2018). Involvement of cystatin C in immunity and apoptosis. Immunology Letters, 196, 80‐90. 10.1016/j.imlet.2018.01.006 29355583 PMC7112947

[acel13253-bib-0062] Zimmer, J. S. , Monroe, M. E. , Qian, W. J. , & Smith, R. D. (2006). Advances in proteomics data analysis and display using an accurate mass and time tag approach. Mass Spectrometry Reviews, 25(3), 450‐482. 10.1002/mas.20071 16429408 PMC1829209

[acel13253-bib-0063] Zimmerman, A. W. , Joosten, B. , Torensma, R. , Parnes, J. R. , van Leeuwen, F. N. , & Figdor, C. G. (2006). Long‐term engagement of CD6 and ALCAM is essential for T‐cell proliferation induced by dendritic cells. Blood, 107(8), 3212‐3220. 10.1182/blood-2005-09-3881 16352806

