# Supplementary figures and images for: Proteomic assessment of serum biomarkers of longevity in older men
Source: Aging Cell. 2020 Oct 20;19(11):e13253. doi: 10.1111/acel.13253 (PMC7681066; doi:10.1111/acel.13253)

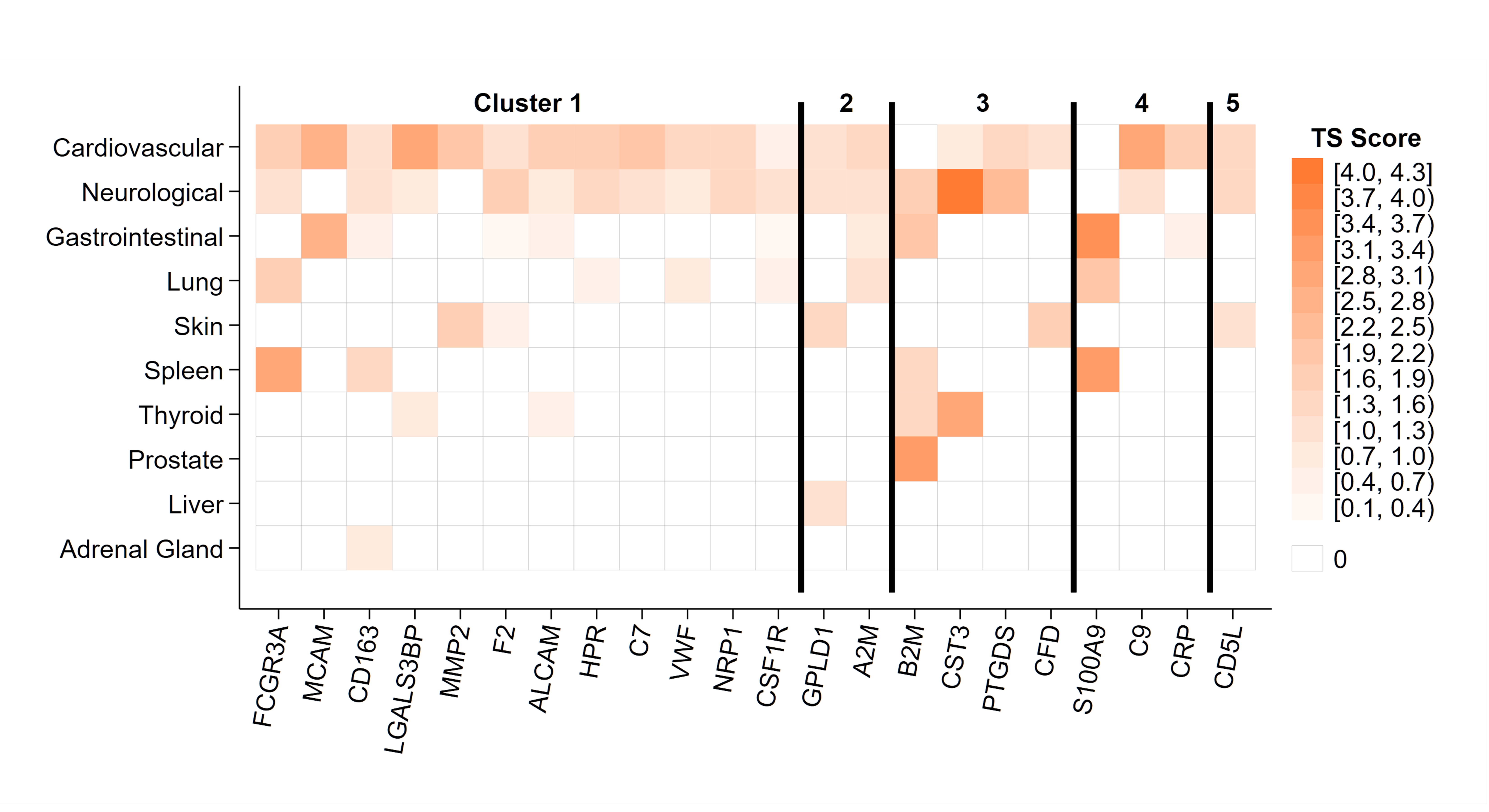

Supplement: Supplementary file 1 [file ACEL-19-e13253-s001.png]

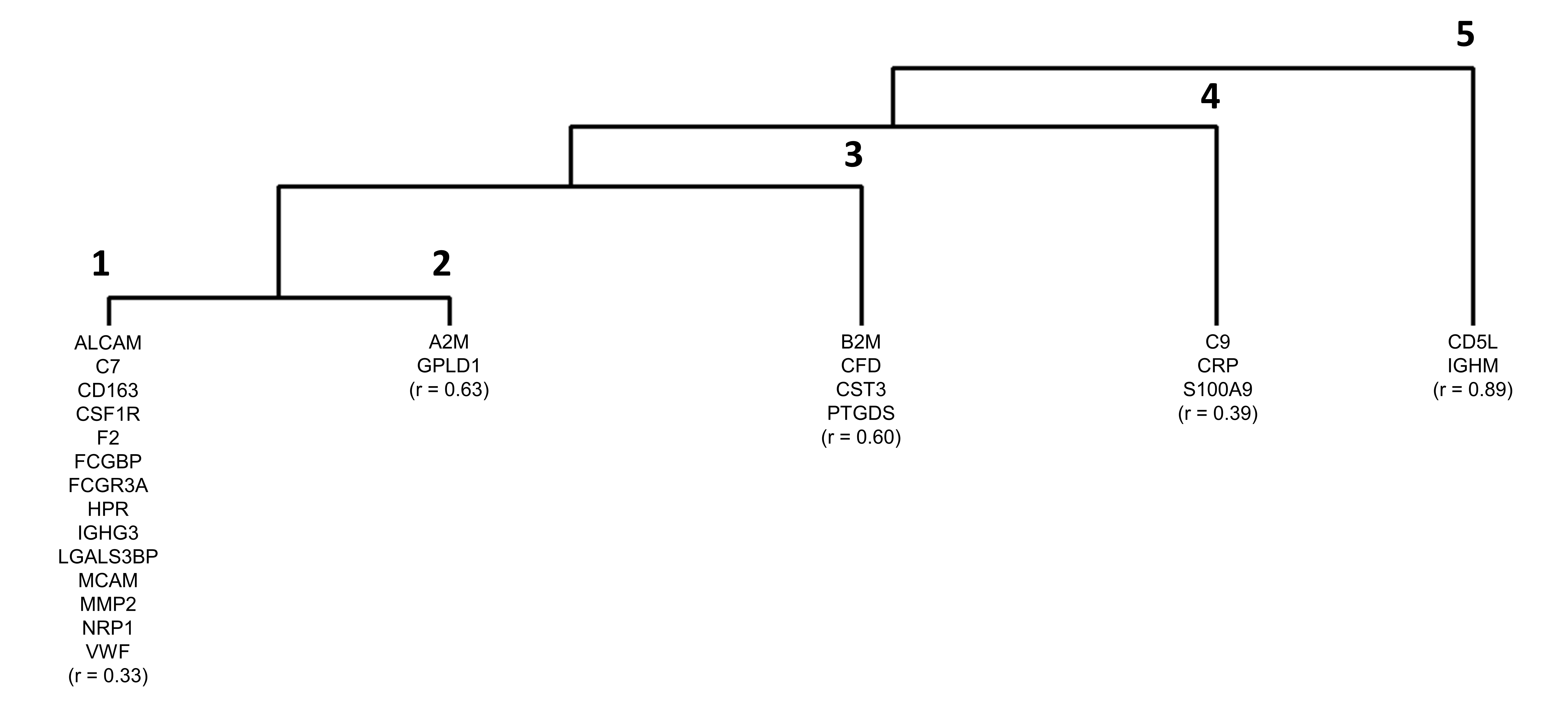

Supplement: Supplementary file 2 [file ACEL-19-e13253-s002.png]

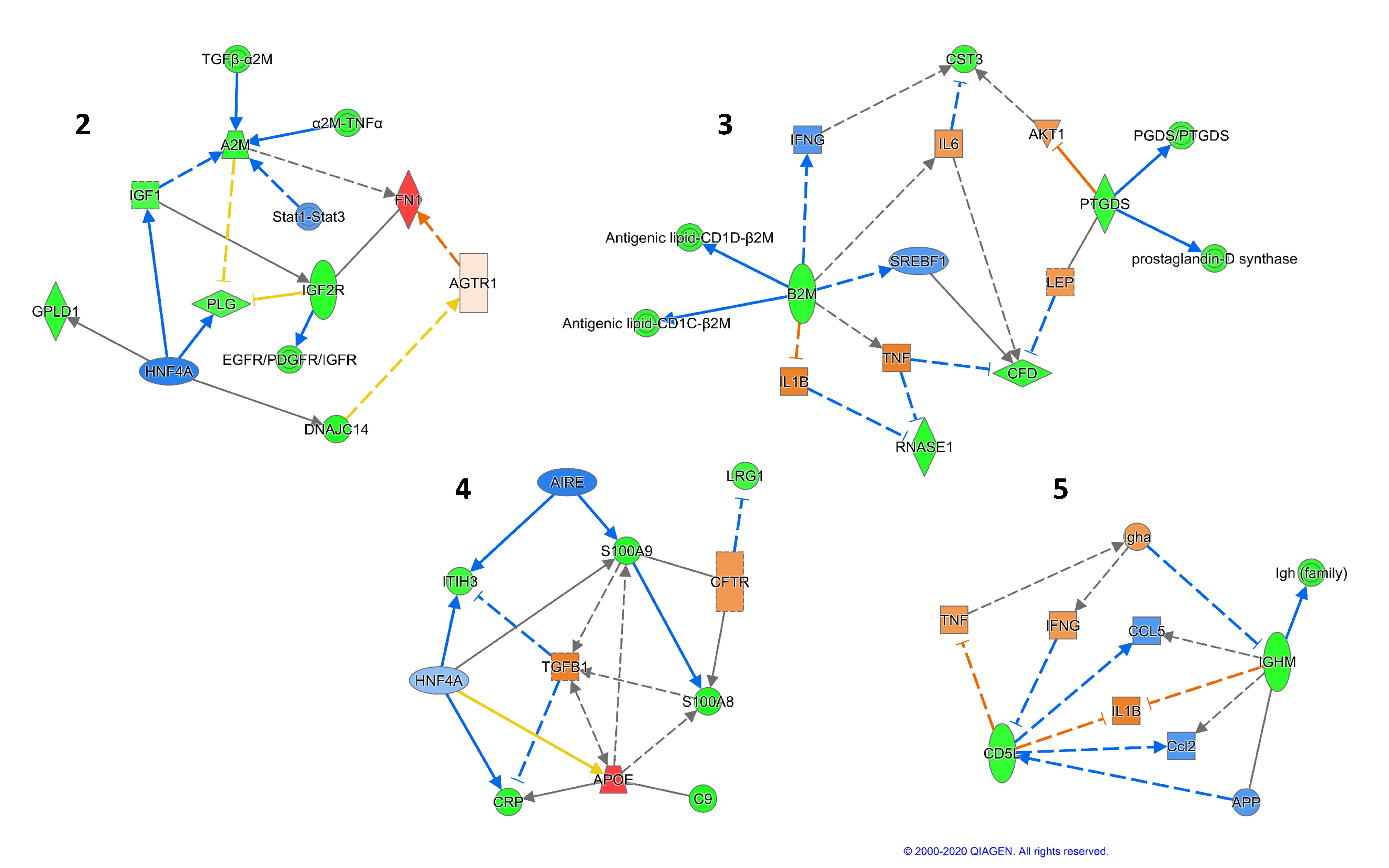

Supplement: Supplementary file 3 [file ACEL-19-e13253-s003.png]

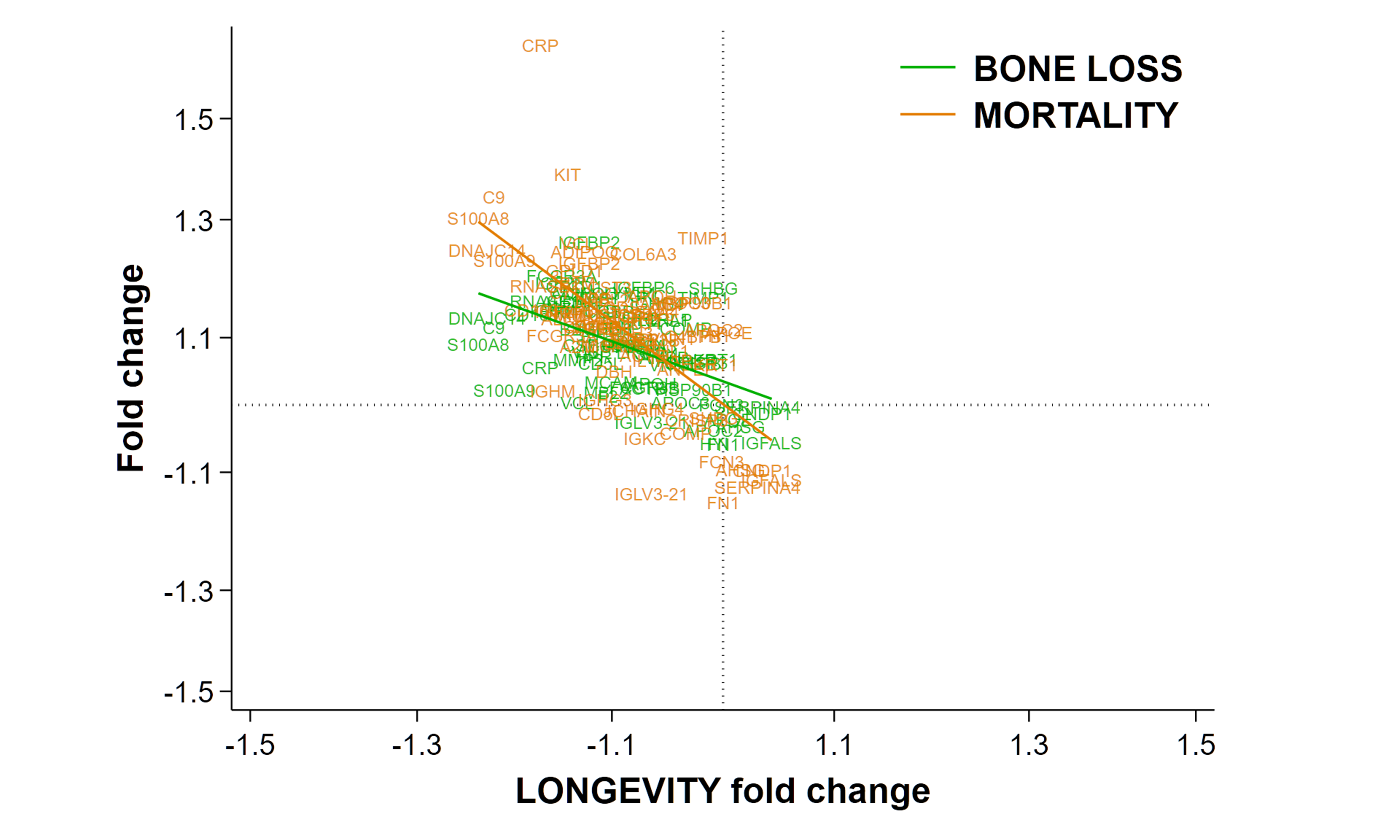

Supplement: Supplementary file 4 [file ACEL-19-e13253-s004.png]
